# Drying Technologies for *Stevia rebaudiana* Bertoni: Advances, Challenges, and Impacts on Bioactivity for Food Applications—A Review

**DOI:** 10.3390/foods14162801

**Published:** 2025-08-12

**Authors:** Shahin Roohinejad, Mohamed Koubaa, Seyed Mohammad Taghi Gharibzahedi

**Affiliations:** 1 Division of Food and Nutrition, Burn and Wound Healing Research Center, Shiraz University of Medical Sciences, Shiraz 71348-14336, Iran; 2 Université de Technologie de Compiègne, ESCOM, TIMR (Integrated Transformations of Renewable Matter), Centre de Recherche Royallieu, CS 60319, 60203 Compiègne CEDEX, France; m.koubaa@escom.fr; 3 Institute of Materials Science, Faculty of Engineering, Kiel University, 24143 Kiel, Germany

**Keywords:** *Stevia rebaudiana* Bertoni, drying methods, food quality, low-calorie sweetener, dietary foods, spray drying

## Abstract

*Stevia rebaudiana* leaves and extracts need to be promptly dried after harvest to prevent microbial activity and preserve their bioactive compounds, including glycosides, flavonoids, and essential oils. Effective drying also reduces moisture and volume, which lowers packaging, storage, and transportation costs. Therefore, innovative drying methods are necessary to maintain stevia’s physicochemical, sensory, and nutritional properties for functional food formulations. This review evaluates various drying technologies for stevia leaves and extracts, including convective hot air, infrared, vacuum, microwave, freeze, and shade drying, and their impacts on product quality and energy efficiency. It also explores the growing applications of dried and extracted stevia in food products. By comparing different drying methods and highlighting the benefits of stevia in these food formulations, this investigation aims to identify future research directions and optimization strategies for utilizing stevia as a natural sweetener and functional ingredient. Convective hot air drying at higher temperatures was found to be the most energy-efficient, though several studies have reported moderate degradation of key bioactive compounds such as stevioside and rebaudioside A, particularly at elevated temperatures and extended drying times. Infrared drying enhanced antimicrobial activity but resulted in lower levels of polyphenols and antioxidants. Vacuum drying effectively preserved anti-inflammatory compounds like flavonoids. Microwave drying presented strong protection of antioxidant activity and superior particle morphology. Freeze drying, while less energy-efficient, was the most effective at retaining antioxidants, polyphenols, and volatile compounds. Shade drying, though time-consuming, maintained high levels of polyphenols, flavonoids, and essential oils. Advanced techniques like spray drying and electrospraying have been reported to enhance the sensory qualities and stability of stevia extracts, making them ideal for food applications such as dairy and baked products, confectionery, syrups, snacks, jams, preserves, and meat products. Overall, stevia not only serves as a natural, zero-calorie sweetener but also contributes to improved health benefits and product quality in these diverse food formulations.

## 1. Introduction

Nowadays, a high percentage of people across the world are facing the problem of diabetes due to changes in lifestyle and genetic predisposition. According to the World Health Organization (WHO), approximately 830 million adults—around 14% of the global adult population—were living with diabetes in 2022, and more than half were not receiving appropriate treatment [1]. Similarly, the International Diabetes Federation (IDF) estimated that 529 million adults aged 20–79 years (11.2%) had diabetes in 2021, a number projected to rise to 643 million by 2030 and 783 million by 2045 [2]. High consumption of added sugars and excess caloric intake are key dietary factors contributing to the development of type 2 diabetes. Although both nutritive and non-nutritive sweeteners are widely used to reduce sugar intake, their long-term metabolic effects remain a topic of ongoing scientific debate. Sweeteners can be broadly categorized into natural and artificial sweeteners. Cane sugar is a natural sweetener with high calories, which is commonly used, while artificial sweeteners such as aspartame and saccharin offer low-calorie options. However, both types have their drawbacks. Excessive consumption of natural sweeteners contributes to obesity and diabetes, and the prolonged use of certain artificial sweeteners (e.g., aspartame and acesulfame potassium) has been subject to public health debate, with some studies suggesting potential associations with adverse effects, such as headaches, gastrointestinal discomfort, or possible carcinogenicity. However, conclusive evidence from human studies remains limited and inconsistent [3,4]. These concerns have led to growing interest in natural, low-calorie sweeteners such as *Stevia rebaudiana*, presenting both sweetness and potential health-promoting bioactive compounds.

*S. rebaudiana* (Bertoni), commonly known as sweet herb, sweet grass, candy leaf, sweet leaf, sugar leaf, or *S. rebaudianum*, is an example of a natural sweetener. It is a dicotyledonous plant (a plant with two seed leaves) belonging to the Compositae (Asteraceae) family. Native to South America, particularly Paraguay, Brazil, and Argentina, stevia was first utilized by the indigenous Guaranì people. Stevia’s sweetness, 300 times higher than sugar, is due to the high content of Steviol glycosides in the leaves. It is calorie- and carbohydrate-free, posing no risk of sudden spikes in blood sugar levels [5,6,7]. Incorporating this ingredient into various food products could help to overcome the aforementioned health problems caused by the consumption of high-calorie natural sweeteners and artificial sweeteners.

**Figure 1:**
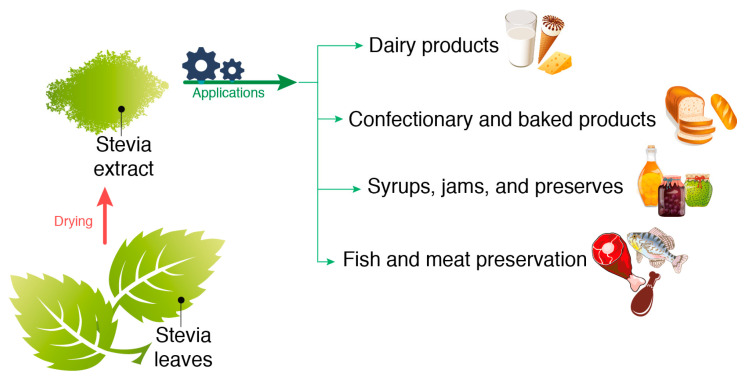
Schematic illustration of stevia leaf extracts in food applications.

Stevia leaves are commonly consumed in different forms, such as fresh, dried, or crushed, as well as through the extraction of their sweet components. However, steviol glycosides are not suitable for certain food applications that involve heating, such as baking, sterilization, and frying, or for applications requiring controlled release of the sweet taste (Figure 1). This is because hydrolysis of part of the molecule can occur, leading to a loss of sweetness. To address this limitation, microencapsulation is often employed for sweeteners to provide protection against high temperatures, enhance fluidity, and prolong the sweetness sensation by controlling release [8,9,10]. The microencapsulation process typically involves preparing an emulsion or dispersion containing the encapsulating agent and the core material (sweetener), which is then dried.

**Figure 2:**
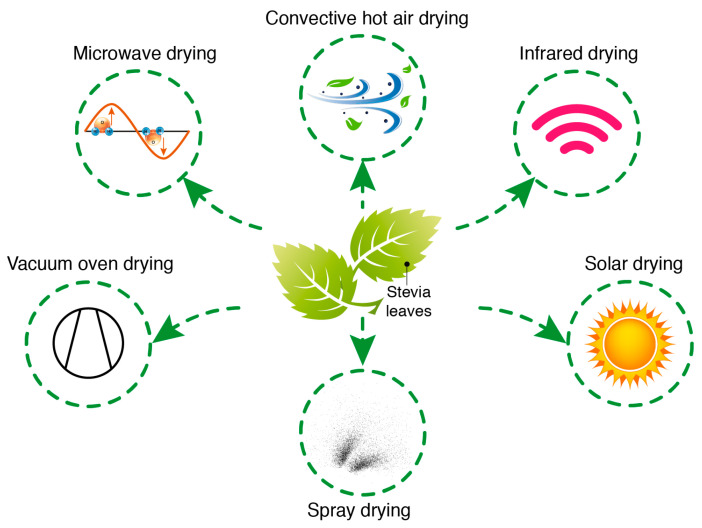
Set of technologies for drying stevia leaves and extracts.

Different drying techniques (e.g., solar drying, shade drying, far-infrared (IR) radiation, medium- and short-wave IR drying, hot air (convective) drying, microwave drying, vacuum oven drying, spray drying, and freeze drying) are commonly used for the microencapsulation of stevia leaves and extracts (Figure 2). These methods are applied to enhance the stability of stevia powder under various environmental conditions, mask off-flavors, and control the release of encapsulated stevia under pre-determined conditions by reducing hygroscopicity and reactivity [11]. The selected drying conditions affect the quality of the stevia powder [12]. Therefore, this review critically compares drying methods for *Stevia rebaudiana* in terms of their effects on bioactive compound retention, drying efficiency, and suitability for various food applications.

## 2. Bioactive Compounds of *Stevia rebaudiana*

*S. rebaudiana* is known for its sweet-tasting compounds, primarily steviol glycosides, which are the bioactives responsible for its sweetness. The main steviol glycosides in *S. rebaudiana* are stevioside (4–13%) and rebaudioside A (2–4%). In addition, several minor glycosides such as Rebaudioside B, Rebaudioside C, Rebaudioside D, Rebaudioside E, Rebaudioside F, dulcoside A, steviolmonoside, steviolbioside, and Rubusoside are present in smaller quantities [8,13]. The concentration of steviol glycosides is influenced by different factors such as cultivar, farming methods, and environmental conditions [14]. These glycosides have distinct sensorial and physicochemical properties. For instance, Rebaudioside A, which contains an additional glucose unit compared to stevioside, is considered superior in terms of sweetness and taste quality, while pure stevioside often has a noticeable bitter aftertaste. The natural ratio of stevioside to rebaudioside A in *S. rebaudiana* leaves is typically around 2, leading to the characteristic bitter aftertaste associated with extracts containing stevioside [8,13,14].

In addition to glycosides, stevia is abundant in ascorbic acid, β-carotene, amino acids (excluding tryptophan), and various minerals such as chromium, cobalt, magnesium, iron, potassium, phosphorus, riboflavin, thiamin, tin, and zinc [15]. Stevia leaves are also known for their richness in phenolic compounds, including vanillic acid 4-HAI-β-D-glucopyranoside, protocatechuic acid, caffeoylquinic acids, dicaffeoylquinic acids, chlorogenic acid, and cryptochlorogenic acid [16] and flavonoids such as apigenin-4-*O*-β-D-glycoside, kaempferol-3-*O*-rhamnoside, quercetin-3-*O*-β-D-arabinoside, quercetin-3-*O*-glucoside, and quercetin-3-*O*-rutinoside [17]. Furthermore, the leaves are a valuable source of carbohydrates, protein, fiber, and antioxidant compounds [14,18].

## 3. Health Benefits and Safety Considerations of Stevia Products

Stevia leaves, which have been used for centuries as a traditional medicine in South America, are increasingly studied for their health benefits [19]. According to JECFA [20], the acceptable daily intake (ADI) of steviol glycosides is 0–4 mg/kg body weight per day. This natural sweetener contains various bioactive compounds that offer a wide array of health benefits. In a recent study, Morissette et al. [21] investigated the effects of chronic administration of two components of *S. rebaudiana* (rebaudioside A and rebaudioside D) on reducing adiposity and hepatic lipid accumulation in an animal model. Another study found that the cookies enriched with stevia powder and extracts led to a reduction in blood glucose levels (4.54% to 7.0%) and an increase in insulin levels (4.27% to 6.13%) compared to the control group [22]. Skeletal muscle plays a crucial role as the primary consumer of insulin-stimulated glucose in the body. However, in type 2 diabetes mellitus, insulin resistance can result in muscle damage. Han et al. [23] reported that the administration of stevia extracts reduced insulin resistance by regulating mitochondrial function and oxidative stress in skeletal muscle.

Several studies have evaluated the anti-inflammatory effects of stevia leaves and extracts [24,25,26]. Farid et al. [27] found a reduction in interleukin (IL)-10, a key anti-inflammatory cytokine, levels by 1.49-fold using 40.5 mg/mL of stevia extract. Although some studies suggest that stevia extracts may modulate cytokine activity, including IL-10, the biological significance of reduced IL-10 levels remains unclear, as this cytokine is typically associated with anti-inflammatory responses. Further research is warranted to clarify the implications of these effects for inflammation control, tissue repair, and autoimmune regulation [28].

Extensive research has been conducted on the antioxidant activity, antibacterial capabilities against a wide range of foodborne pathogens (such as *Escherichia coli*, *Salmonella typhimurium*, *Pseudomonas aeruginosa*, *Staphylococcus aureus*, and *Listeria monocytogenes*), and anticancer properties of stevia leaves and extracts [19,29,30,31,32,33,34,35,36]. Stevia’s antioxidant activity has been reported to mitigate the spread of free radicals, including superoxides, which are known to cause DNA damage [37]. These antioxidant properties can also inhibit cell damage, potentially reducing the risk of developing carcinogenesis and tumors. Chakma et al. [38] explored the impact of different stevia extraction methods on antioxidant and antibacterial properties and found that the most effective extraction method was using 80% aqueous ethanolic extracts as a solvent, yielding the highest activity. The synthesized stevia nanoparticles exhibited notable characteristics, which exhibited the highest antioxidant activity (~75 mg gallic acid equivalent (GAE)/g extract) when chloroform/methanol (2:1 *v*/*v*) was used as a solvent. Furthermore, these nanoparticles demonstrated significant antibacterial activity against foodborne pathogens such as *E. coli* and *S. aureus*, as well as superior anticancer activity against the lung cancer cell line of A549 [39].

## 4. Drying Technologies of *Stevia rebaudiana* Leaves

Drying is a widely utilized method for preserving plant materials worldwide [40]. This process enhances food stability and extends shelf life by significantly reducing water content and inhibiting microbial growth as well as preventing certain physical and biochemical reactions that could degrade product quality. Moreover, drying leads to a considerable reduction in weight and volume, thereby reducing packaging, storage, and transportation costs [14,16,41,42,43]. Although low drying temperatures (30 °C to 50 °C) are recommended for drying of medicinal plants, drying at such temperatures significantly prolongs the processing time and consequently reduces the capacity of the drying instrument. Selecting the appropriate drying method is essential to preserve the characteristics of food and minimize any nutritional and physicochemical changes [4,40].

Similar to other plant materials such as medicinal herbs and aromatic plants, drying is a critical post-harvest handling process for freshly harvested stevia leaves. The high moisture content of fresh stevia leaves, around 80%, makes them susceptible to oxidative blackening, resulting in the degradation of bioactive compounds and storage challenges. The drying process reduces the moisture content, enhancing storage stability by inhibiting microbial growth, and maintaining stable quality [16,44]. Furthermore, reducing the moisture content decreases the volume requirements for packaging and storage space, thereby lowering transportation costs.

The selection of a drying method is influenced by various factors, including the plant species, the moisture content of the leaves, and the nature of the active compounds (such as steviol or antioxidants), which can be affected by the drying temperature. However, the costs associated with the drying method should also be considered [14]. The effect of different drying methods on the physicochemical, sensory, and nutritional properties of stevia leaves and extracts is discussed below (Table 1).

### 4.1. Solar Drying

Drying stevia plants and leaves is commonly accomplished by exposing them to direct sunlight. Solar dryers are more energy-efficient compared to other industrial dryers because they use available solar energy. This drying method offers additional advantages, including space efficiency, higher product quality, environmental friendliness, and reduced CO_2_ emissions [44,53,54]. However, this approach has some drawbacks, including inadequate drying, susceptibility to rain damage, and vulnerability to pests, affecting the final product’s quality, leading to color changes and the loss of valuable nutrients [19,53].

To dry stevia effectively, branches should be exposed to sunlight for 4–6 h and harvested when they reach 10–12% humidity [7]. During open-sun drying, branches and leaves are spread out without overlapping on surfaces such as cloth or plastic. If not fully dried by sunset, they are stored indoors, and the process is repeated daily until leaves are completely dry, then separated from stems. Stevia leaves can achieve a humidity reduction from 80% to 10% within 9 to 10 h under low humidity and thin layering conditions. In solar drying, heat is transferred primarily through convection and radiation, and mass transfer occurs via diffusion of water vapor to the surface. However, the relatively low and fluctuating temperatures can lead to incomplete drying or uneven internal moisture gradients. These conditions may cause surface hardening (case hardening), which inhibits further moisture escape and promotes oxidative degradation of thermolabile compounds like steviol glycosides and polyphenols [44]. However, if the leaves are not dried quickly after cutting, their quality deteriorates due to oxidation, with up to a third of the stevioside content being lost after three days of exposure [12].

Solar drying technologies vary widely, with common types including direct cabinet dryers, indirect dryers often using solar air heaters, and mixed dryers that combine direct and indirect methods. Several studies evaluated the feasibility of using this method to dry stevia plants and leaves. For instance, Castillo-Téllez et al. [7] studied the characteristics of dehydrated stevia leaves using various solar drying technologies. They compared direct drying (cabinet type) with natural and forced convection to indirect drying, with air heated by a solar water heating system. Both methods were found to be technically feasible for drying stevia leaves. Indirect solar drying offered benefits such as moderate drying times, better control over operating conditions, and enhanced protection against temperature effects than direct solar radiation exposure. Drying kinetics were similar for both methods, with equilibrium achieved between 240 and 270 min. Temperature was found to be the most significant factor affecting color degradation. Using a shadow-mesh cover in the direct solar dryer improved leaf quality and reduced discoloration. The Weibull and Two-term exponential models were identified as the best fit for the experimental results of direct solar drying, while the Weibull model was optimal for indirect solar drying [7,55].

Another study compared the drying of stevia leaves using a newly developed mixed-mode forced convection solar dryer (MFSCD) and open sun drying (OSD) [12]. The MFSCD achieved a final equilibrium moisture content of 0.053 d.b. in 330 min, whereas the OSD method required 870 min to reach the same moisture content level. The MFSCD achieved an overall dryer efficiency of 33.5% and an average exergy efficiency of 59.1%. Solar-dried samples had higher antioxidant and flavonoid content and better color preservation compared to OSD samples. Sensory analysis showed that solar-dried stevia leaves received higher flavor, aroma, and taste scores than OSD samples. Sensory analysis, conducted with 15 untrained panelists using a balanced 9-point hedonic scale, showed that solar-dried stevia leaves received significantly higher scores for flavor, taste, texture, and overall acceptance [12].

Hidar et al. [44] investigated the effects of solar convective drying on the kinetics and quality of stevia leaves from Morocco. Drying was conducted at different temperatures (50, 60, 70, and 80 °C) and air flows (300 and 150 m^3^/h). Effective moisture diffusivity ranged from 5.07 × 10^−11^ to 3.14 × 10^−10^ m^2^/s, with an activation energy of 37.81 kJ/mol. They observed an increase in the effective moisture diffusivity at higher drying temperatures and airflows. While the study demonstrated the technical feasibility of solar drying for stevia, it also highlighted that those drying conditions, particularly temperature and airflow, significantly impact the quality of the leaves. Higher temperatures and airflows decreased chlorophyll, total phenolic, and flavonoid content. Microstructural deformations occurred at high temperatures (e.g., 80 °C) and air flows due to rapid water diffusion and evaporation. The drying kinetics of stevia leaves using a convective dryer at different air velocities (2, 3, and 4 m/s) and temperatures (45 °C, 55 °C, and 65 °C) was recently studied [43]. The highest drying rate was achieved at 65 °C and an air velocity of 4 m/s, with a rate of 0.05 kg water/kg (dry matter min). The experimental data were fitted to the Page model, which showed a good fit (correlation coefficients (R^2^) > 0.9994). Equations were derived to correlate the model’s variables with air velocity and temperature. The results indicated that total color difference (ΔE) and glucoside values increased with higher temperatures and air velocities [43].

### 4.2. Infrared Drying

Infrared (IR) drying is a technique that employs IR radiation to extract moisture from a variety of materials. Unlike conventional methods that depend on convection or conduction, IR drying directly heats the material, leading to quicker and more effective drying [16,45]. IR drying methods utilize specific wavelengths of IR radiation. These include near-IR drying, medium- and short-wave IR drying (MSWID), and far-IR drying with wavelengths ranging from 0.7 to 1.5, 1.5 to 3.0, and 3.0 to 1000 μm, respectively.

**Figure 3:**
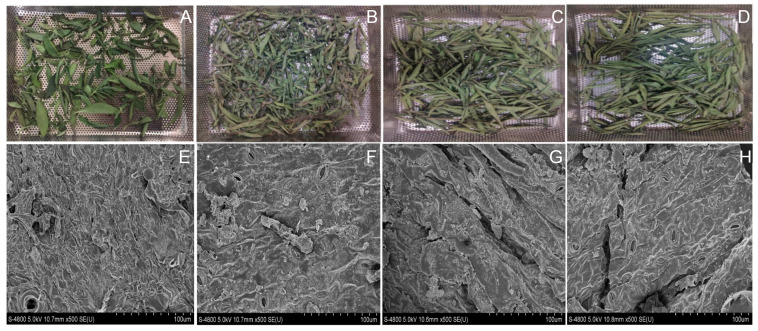
Photographic (**A**–**D**) and scanning electron microscopic (SEM; **E**–**H**) images of *S. rebaudiana* leaves under different drying conditions: (**A**,**E**) Fresh samples. (**B**,**F**) Natural air drying. (**C**,**G**) Hot air drying at 55 °C. (**D**,**H**) Far-IR drying at 55 °C. Adapted and reprinted from Huang et al. [45] with permission from Elsevier.

Several studies have assessed the impact of this method on drying stevia leaves. For instance, far-IR drying was used to dry stevia leaves, exploring drying rate, kinetics, glycoside content, and surface structure at different temperatures (45–65 °C) and irradiation heights (60–300 mm). It was found that far-IR drying minimized color changes and enzymatic browning more effectively than natural air drying (Figure 3), while its color impact was comparable to hot air drying [45]. They also displayed that far-IR drying resulted in more micropores and minimal shrinkage due to rapid heating and synchronized heat and water migration, preserving the structural integrity of *S. rebaudiana* leaves. In contrast, hot air drying caused severe shrinkage and quality degradation, including discoloration due to chlorophyll loss, reduced steviol glycoside content, and diminished antioxidant activity (Figure 3). Moisture rate decreased and drying rate increased significantly with higher temperatures and lower irradiation heights. The Weibull model fit well (R^2^ > 0.99), with estimated water diffusion coefficient D_cal_ ranging from 0.8418 × 10^−4^ to 2.9453 × 10^−4^ m^2^/s, and effective diffusion coefficient D_eff_ ranging from 0.8359 × 10^−6^ to 3.6291 × 10^−6^ m^2^/s. The activation energy was 28.79 kJ/mol as per the Arrhenius equation. Increasing the drying temperature from 45 °C to 55 °C increased the levels of stevioside and rebaudioside A. Far-IR drying also increased the number of micropores on and within the material surface, enhancing the quality of the dried product [45]. The micropore formation observed during IR drying enhances moisture diffusion by increasing internal surface area and reducing resistance to water transport. This rapid moisture removal helps prevent thermal degradation of steviol glycosides and polyphenols, which are otherwise sensitive to prolonged heat exposure [45]. Bakhshipour et al. [5] investigated the impact of an IR-assisted continuous-flow hybrid solar dryer on stevia leaves, varying inlet air temperatures (30 °C, 40 °C, and 50 °C), air inlet velocities (7, 8, 9 m/s), and IR lamp input powers (0 W, 150 W, and 300 W). They found that both inlet air temperature and IR lamp input power significantly affected drying time (*p* < 0.05). The study compared various models for predicting moisture ratio during drying, including mathematical models, artificial neural network (ANN) models, and Adaptive Neuro-Fuzzy System (ANFIS) models, with ANN being the most accurate. Among the mathematical models, the Midilli model demonstrated the best fit. They concluded that artificial intelligence models effectively predict the drying kinetics of stevia leaves in this type of dryer.

Previous studies have explored the potential of MSWID for drying various fruits and vegetables. These investigations found that the drying efficiency, which significantly impacts product quality, depends on factors such as wavelength, applied temperature, material absorption behavior, sample thickness, and the distance between the MSWID device and the sample [56,57]. Ai et al. [16] recently compared the physicochemical properties of stevia leaves dried using MSWID and other methods (e.g., hot air impingement drying, hot air drying with temperature and humidity control, and shade and sun drying). MSWID significantly reduced drying time by 15.38% and better preserved nutrients, particularly steviol glycosides and chlorogenic acids, attributed to the formation of a distinct microporous structure. Furthermore, medium-wave IR drying was superior to short-wave IR in retaining steviol glycosides and color while improving drying efficiency. An observational association between greenness and chlorogenic acid content was reported, although this relationship was not statistically validated in the original study. For optimal drying results, medium-wave IR drying at 60 °C was recommended due to its highest comprehensive score (0.723), considering factors such as drying time, energy consumption, and product quality.

### 4.3. (Convective) Hot Air Drying

Convective hot air drying is a popular method for drying a wide range of agricultural goods in field, industrial, and commercial settings due to its ease of use, low cost, and ability to provide consistent and efficient drying rates across a wide temperature range. This method extends shelf life and is beneficial in the development of processed foods. It is a common postharvest technology for extending shelf life, preserving quality, and improving stability by reducing moisture content. Proper management of the drying process can lead to a higher yield of high-quality products. Monitoring control variables, such as air-drying temperature, can minimize the undesirable effects of hot air drying and improve the nutritional value of the food product [6,17,58,59]. Convective hot air drying relies on external heat conduction and internal moisture diffusion. As temperature increases, water vapor pressure gradients intensify, accelerating internal moisture migration. However, elevated drying temperatures can collapse plant cell walls, disrupt microstructure, and impair mass transfer. This structural collapse may hinder the release of intracellular steviol glycosides and antioxidants or expose them to oxidative degradation, depending on the balance between enhanced release and thermal breakdown [6,7,17,18].

In a recent study by Kalsi et al. [6], the drying of stevia leaves was investigated using a lab-scale convective hot-air dryer with temperatures ranging from 30 to 80 °C. To better understand the drying kinetics, the drying behavior and the development of predictive models were analyzed using mathematical, ANNs, and ANFIS approaches. The physicochemical properties of the dried leaf powders were also analyzed after drying. The results showed that the ANFIS model, with an R^2^ of 0.9998, provided a more accurate prediction of the drying kinetics compared to the mathematical and ANN models. The convective drying process significantly impacted the color properties of the dried leaves, including L*, a*, b*, hue angle, and chroma values. Furthermore, increasing the drying temperature from 30 to 80 °C led to a 50.90% decrease in water activity (aw), a 10.10% decrease in tapped density, and a 23.26% increase in water solubility index. The hygroscopicity increased by 32.93%, particle density by 54%, and bulk porosity by 10.59% in dried leaf powders. Ascorbic acid and antioxidant activity decreased with increasing temperatures, while total phenolic content (TPC) increased up to 50 °C. The bulk density of the dried samples remained largely unchanged with increasing temperature, while the flowability of the stevia powder improved.

In a study by Lemus-Mondaca et al. [17], the impact of different temperatures (30, 40, 50, 60, 70, and 80 °C) at a constant air velocity (2.0 m/s) on stevia leaves’ bioactive compounds, antioxidant capacity, sweetness, and mineral content was investigated. The study found that vitamin C content decreased proportionally with drying temperature. Phenolics and flavonoids showed an increase when dried at temperatures below 50 °C. The highest antioxidant activity was observed at 40 °C, with a stronger correlation with phenolic and flavonoid contents. The study also determined the content of eight natural sweeteners in stevia leaves and found an increase in seven of the sweeteners, excluding steviolbioside, at drying temperatures up to 50 °C. However, the content increase of sweeteners was not significant at temperatures between 60 and 80 °C.

### 4.4. Microwave Drying

In recent years, microwave drying has become increasingly popular due to its ability to reduce drying time, ensure uniform energy dissipation, enhance energy recovery, and improve final product quality. Microwaves, with frequencies ranging from 300 MHz to 300 GHz, cause polar particles in the material to rotate, generating friction and producing thermal energy. This creates significant volumetric heat and a gradient of internal vapor pressure, moving moisture from the core to the surface. The internal vapor pressure gradient facilitates fast moisture transport without requiring high external temperatures, thereby limiting the thermal degradation of heat-sensitive compounds like ascorbic acid and rebaudioside A. Unlike traditional drying methods that transfer heat from the surface to the core, which prolongs the drying process, microwave drying is more efficient and faster, leading to better food product quality and lower energy consumption [46,60,61,62].

Kalsi et al. [46] investigated the impact of different microwave power levels (180 to 900 W) on the drying process of stevia leaves. The drying behavior was assessed using five thin-layer models, and the semiempirical Page model demonstrated a strong fit with an R^2^ value exceeding 0.997. Increasing microwave power resulted in an increase in the effective diffusivity from 3.834 × 10^−11^ to 1.997 × 10^−10^ m^2^/s. The specific energy consumption initially increased to 9.77 MJ/kg at 320 W and then decreased. The moisture ratio during microwave drying was predicted using a multilayer feed-forward (MLF)-based ANN. A high R^2^ value of 0.999 was achieved by the ANN model with 15 neurons in one hidden layer, indicating the effectiveness of this model in predicting drying kinetics. Furthermore, significant differences in color properties were observed between fresh and dried samples, except for the hue angle, which remained consistent regardless of the microwave dryer’s power output. At the 720 W power level, the highest stevioside (11.84 mg/g) and rebaudioside A (7.11 mg/g) contents were obtained, along with approximately 86% retention of ascorbic acid. Additionally, microwave drying at 900 W resulted in the highest total phenol content (56.98 mg GAE/g) and antioxidant capacity (74.22%) in the dried samples [46].

### 4.5. Vacuum Oven Drying

A vacuum oven is a specialized appliance designed to operate under reduced atmospheric pressure, typically below standard atmospheric pressure. This unique setting enables gentler drying at lower temperatures compared to conventional ovens, a concept supported by the principles of thermodynamics. Nurhad et al. [63] showed that under low absolute pressure (vacuum conditions), the evaporation of water vapor can occur at lower temperatures compared to normal conditions. This reduction in pressure effectively lowers the boiling point of liquids, which is advantageous for drying delicate materials or substances prone to thermal degradation. Because the drying occurs below typical boiling points, the risk of Maillard reactions and glycoside decomposition is minimized, preserving bioactive integrity [46]. Vacuum drying has been shown to mitigate non-enzymatic browning, a process influenced by temperature [64]. Research indicates that vacuum dryers tend to produce more amorphous forms compared to crystalline forms [63].

A few studies have examined the use of vacuum oven drying for stevia leaves and extracts. These studies have investigated the potential of integrating vacuum drying while adding a coating below the glass transition temperature of the stevia solution to improve the drying efficiency. Fishi et al. [37] investigated the impact of different ratios (1:1, 1:2, and 1:3) of Arabic gum and maltodextrin on the physicochemical properties of vacuum-dried stevia extract powder. Stevia leaves were extracted using the microwave-assisted extraction method. The addition of maltodextrin increased the powder’s hygroscopicity and angle of repose, while Arabic gum reduced both properties. Arabic gum was more effective in preserving bioactive compounds, whereas higher levels of maltodextrin decreased the bioactivity of the stevia extract powder after vacuum drying. The stevia extract powder produced using a 1:1 ratio of Arabic gum to maltodextrin exhibited the lowest hygroscopicity (1.40 × 10^−4^ g H_2_O/g) and angle of repose (31.35 ± 0.4°), as well as the highest total phenolic activity (1.52%) and stevioside content (0.073%) [37].

### 4.6. Spray Drying

Among the various encapsulation methods, spray drying is a widely used, simple, and cost-effective technology in both the food and pharmaceutical industries. This method transforms liquids into powders through a three-step process: atomizing the liquid, drying the resulting droplets, and recovering the powder [65]. The widespread use of spray drying for microencapsulation can be attributed to the availability of spray-drying equipment, low operational expenses, a wide variety of encapsulating agents, and the stability of the final product [8,11]. The rapid moisture removal in spray drying, driven by a high temperature gradient and fine droplet atomization, reduces thermal exposure time, thereby limiting degradation of heat-sensitive compounds. Moreover, encapsulating agents form a protective matrix that entraps bioactives within an amorphous structure, enhancing their thermal stability and resistance to oxidative degradation [59].

Several studies have evaluated the feasibility of using spray drying to dry aqueous extracts of stevia. Recently, Chaparro-Hernández et al. [49] investigated the impact of spray drying process variables, including inlet air temperature (160–180 °C) and flow rate (2–3 kg/h), on the concentration of total polyphenolic compounds, total flavonoids, and antioxidant activity of stevia aqueous extract. The application of spray drying reduced the polyphenolic compounds and antioxidant activity in all process conditions. Compared to the fresh aqueous extract, the lowest phenolic concentration (38.69%) and antioxidant activity (3.76%) were observed in the samples treated at 160 °C and a flow rate of 2 kg/h. They also observed significant changes in the polyphenolic profile when spray drying was used at 200 °C and a flow rate of 2 kg/h. Fourteen polyphenolic compounds including phenolic acids, flavonols, flavones, dihydroflavones, and isoflavones detected in the aqueous extract were reduced to one phenolic acid under those conditions. Using spray drying at a lower temperature of 160 °C and a higher flow rate of 3 kg/h was found to have the least effect on altering the phenolic compound profile [49].

In a study by Zorzenon et al. [51], the potential of microencapsulating a stevia fraction in a maltodextrin matrix (DE 17–19) was explored using a spray-drying process to allow for higher dosages. The findings showed a microencapsulation efficiency of 84%. The microcapsules exhibited solubility three times higher than the non-encapsulated stevia fraction. Moreover, the microcapsules displayed increased hygroscopicity and reduced moisture content compared to the free stevia fraction. Chranioti et al. [8] studied the encapsulation of steviol glycosides from stevia leaves using spray drying to reduce bitterness and improve their sensory properties. They used a blend of maltodextrin and inulin (80:20 ratio) and tested three steviol glycoside levels (1.5%, 2.5%, and 3.5%). The formulation with 2.5% steviol glycosides in total solids had the most favorable sensory and quality characteristics, making it the best option for enhancing steviol glycoside properties. Martono et al. [3] conducted a study to clarify and encapsulate the water extract of stevia using a spray-drying method. Acid-activated kaolin was used for clarification, which reduced the green pigment by 95% and the yellow pigment by 65%. The stevioside and rebaudioside A contents were found to be 0.83% and 2.25%, respectively. The final products had a moisture content of 9.8% and hygroscopicity of 12.91%. The organoleptic results showed a reduced sweetness level in the encapsulated products.

## 5. Comparison of Drying Technologies for Stevia Leaves and Extracts

### 5.1. Stevia Leaves

Several studies have compared the effects of different drying techniques for encapsulating stevia leaves and extracts (Table 2). For instance, Lemus-Mondaca et al. [66] evaluated the effects of different drying methods on stevia leaves, including convective hot air, IR, and vacuum drying at 40, 60, and 80 °C. Microstructural analysis showed that convective hot air drying best-preserved leaf characteristics. In terms of energy efficiency, convective drying at 80 °C showed the lowest specific energy consumption (61.86 kWh/kg) and highest efficiency (8.5%). The study signified that drying methods remarkably impact the thermophysical properties and energy efficiency of dried stevia leaves, presenting insights into optimizing drying processes [66,67]. Lemus-Mondaca et al. [19] investigated the effects of seven drying methods (freeze drying, shade drying, sun drying, IR drying, vacuum drying, microwave drying, and convective drying) on the quality of stevia leaves. Polyphenols and antioxidant capacity increased in all dried samples, with freeze drying and shade drying yielding the highest values, and IR drying the lowest. IR and convective drying showed longer antimicrobial inhibition periods. The strongest anti-inflammatory effects were seen in vacuum- and microwave-dried samples. Furthermore, microwave-dried, sun-dried, and shade-dried stevia were most effective against TPA-induced inflammation. This study demonstrates how different drying methods affect the bioactive compound content and activity in stevia leaves [19].

The effects of various drying methods (e.g., hot air drying at 100 °C and 180 °C, freeze drying, and shade drying) on steviol glycosides (stevioside, dulcoside A, rebaudioside A, and rebaudioside C) and antioxidants in stevia leaves were previously investigated [13]. Stevioside, the major glycoside in fresh leaves (81.2 mg/g), was significantly reduced in all drying methods, with shade drying causing the least reduction. Hot air drying at 180 °C was the most effective for enhancing the content of phenols, flavonoids, and total antioxidants. The study concluded that the optimal drying method depends on the intended use of stevia leaves and identified hot air drying at 180 °C as the best overall choice [13]. The effects of freeze drying and gamma irradiation (1 kGy) on the moisture sorption behavior and thermodynamic properties of stevia leaves under various storage temperatures (30 °C, 40 °C, and 50 °C) have been recently studied by Hidar et al. [68]. Freeze-dried leaves exhibited higher moisture sorption capacity and surface area due to their porous structure, indicating a need for careful packaging to prevent moisture uptake. Gamma irradiation also increased moisture adsorption compared to untreated leaves. All stevia samples maintained water activity below 0.4, suggesting good biochemical and microbial stability during storage. These findings support both treatments as viable preservation methods, with considerations for optimal storage conditions [68].

Gasmalla et al. [69] examined the effects of three drying methods, including sun, oven, and microwave drying, on the nutritional composition of stevia leaves. Compared to the other drying methods, the heavy metal analysis revealed that the sun-dried samples had the highest levels of lead (4.77 µg/g), cadmium (0.49 µg/g), and arsenic (0.30 µg/g). Another study investigated the impact of sun, oven, and microwave drying methods on the volatile compounds, thermal stability, and surface morphology of stevia leaves [70]. The results showed that the morphology of the dried leaf powders depended on the drying technique: microwave drying resulted in even, regular, and compact particles, while sun-dried and oven-dried particles had angular, brick-like shapes. The choice of drying method was also found to have a significant impact on the structural properties, thermal stability, and volatile compound profile of the stevia leaves [70].

Al-Amrani et al. [14] compared the effects of eight different drying methods, including solar drying, shade drying, oven drying at 40 °C, 50 °C, and 60 °C, and microwave drying for 1, 2, and 3 min, on the total glycoside composition in the leaves and branches of stevia. In leaves, the highest dry matter percentage was observed in samples treated by shade drying and microwave drying for 2 min, while solar drying and oven drying at 50 °C resulted in the best total glycoside sweetener composition. Specifically, the highest stevioside content in leaves was found with oven drying at 50 °C (5.32%), microwave drying for 3 min (5.29%), and solar drying (5.28%), while the highest rebaudioside A content was recorded under microwave drying for 1 min (2.77%) and solar drying (2.58%). For the branches, the highest dry matter percentages were achieved with shade drying and microwave drying for 1 min, but the highest percentage of total steviol glycosides (stevioside and rebaudioside A) was obtained through oven drying at 60 °C (stevioside: 0.655%, rebaudioside A: 0.355%) and microwave drying for 2 min (stevioside: 0.595%, rebaudioside A: 0.350%), followed by solar drying and oven drying at 50 °C [14].

The effects of different drying methods (i.e., hot air drying, freeze drying, and shade drying) on flavonoid, phenolic, and volatile compounds in stevia leaves were previously studied by Periche et al. [71]. Each drying method was found to differently influence the antioxidant and volatile compound profiles of the dried stevia leaves. Freeze drying and shade drying produced the highest levels of 2-hexenal, hexanal, and α-pinene, while hot air drying yielded the highest levels of tetrahydrofuran and α-pinene. Shade drying resulted in the highest content of volatile compounds, whereas freeze drying generally led to higher concentrations of most flavonoids and phenolic acids, although some of these compounds were more abundant following hot air drying. Periche et al. [71] reported that freeze drying preserved the highest concentrations of most phenolic compounds, including chlorogenic acid (191.84 mg/100 g), coumaric acid (91.35 mg/100 g), and sinapic acid (178.56 mg/100 g), with statistically significant differences (*p* < 0.001) compared to air and shade drying. Shade drying resulted in the highest total volatile content (e.g., 2-hexenal: 21.09 µg/g), while hot air drying produced higher levels of certain flavonoids such as 4-methoxybenzoic acid (26.28 mg/100 g), than freeze drying (7.48 mg/100 g). Although freeze drying is widely regarded as the most effective method for preserving thermolabile bioactives in *S. rebaudiana*, one study cited in Table 2 reported the highest total phenolic content following hot air drying at 180 °C. This may be due to thermal breakdown of cell walls and enhanced release of bound phenolic compounds during high-temperature exposure. However, given the potential for degradation of sensitive compounds under such conditions, the suitability of hot air drying at 180 °C should be considered concerning the specific intended application, such as maximizing phenolic content versus preserving antioxidant or glycoside stability. Such findings highlight the complexity of plant matrix responses and suggest that optimal drying conditions may vary depending on the target compound. Moguel-Ordóñez et al. [72] investigated the drying characteristics of stevia leaves using radiation, convection, sun, and shade-drying treatments. Convection- and shade-dried samples exhibited better color and the highest total pigment contents. Extracts from leaves dried using convection- and shade-drying showed higher Trolox equivalent antioxidant capacities and ferric-reducing power. Among the different drying techniques, convection drying and shade drying were found to be the least aggressive. Abdul Halim et al. [15] evaluated the effects of microwave and freeze drying on antioxidant activity, TPC, and steviol glycosides in stevia leaves. Microwave drying yielded the highest TPC (53.95 mg GAE/g) and antioxidant activity (89.16%), while freeze drying resulted in the highest stevioside content (176% increase) and rebaudioside A (0.0335 mg/g), with statistically significant differences compared to other methods.

### 5.2. Stevia Extracts

**Figure 4:**
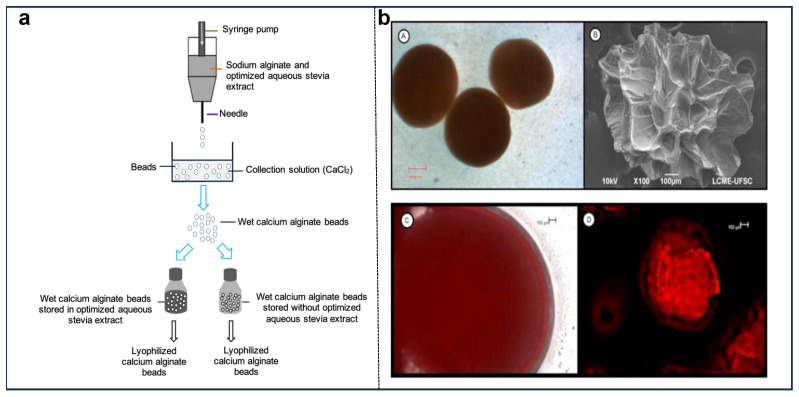
(**a**) Schematic representation of the encapsulation procedure of the optimized aqueous stevia extract with sodium alginate. (**b**) Microscopic and SEM images of calcium alginate beads: (**A**) wet (bar = 500 µm) and (**B**) lyophilized (bar = 100 µm); confocal microscopy images of (**C**) wet (bar = 100 µm) and (**D**) lyophilized (bar = 100 µm) beads encapsulating aqueous S. rebaudiana leaf extract. Retrieved (Figure 4a) and reprinted (Figure 4b) from Arriola et al. [74] with permission from Elsevier.

A few studies also compared the effects of different drying techniques for encapsulating stevia extract. Oikonomopoulou et al. [65] investigated the impact of different encapsulation methods, such as spray drying and electrospraying, on the quality of stevia extract powder and the reduction of its glycoside aftertaste. Maltodextrin-inulin (50:50) was used as the matrix for spray drying, while zein was used for electrospraying. The study reported encapsulation efficiencies of 66.37% for spray drying and 67.47% for electrospraying. SEM images revealed that the resulting particles were spherical, with mean diameters of 1.35 μm for electrosprayed particles and 6.02 μm for spray-dried particles. Differential scanning calorimetry (DSC) showed that the glass transition temperature (T_g_) of the encapsulated structures was lower than that of the pure matrices, with T_g_ being influenced by the processing conditions. Sensory evaluations conducted by trained panelists in an ISO 17025-accredited laboratory indicated that both electrospraying and spray drying significantly reduced the bitterness of stevia extracts, improving their sensory acceptability for incorporation into innovative food formulations. Another study by Özyiğit et al. [73] examined the effects of various drying methods (i.e., sun, shade, air conditioning, and oven) on the chemical composition and bioactivity of stevia extract. Antioxidant activity was highest in the extracts dried in the shade and in the oven. However, air-conditioned drying resulted in the highest concentration of 2-tetradecyl acrylate and showed strong antibacterial activity, as well as cytotoxic effects evaluated via XTT assay against MDA-MB-231 (human breast carcinoma) and L929 (mouse fibroblast) cell lines, suggesting potential selective toxicity toward cancer cells. Chranioti et al. [11] compared the effects of different drying methods, including spray drying, freeze drying, and vacuum oven drying on reducing bitterness and improving the properties of steviol glycosides. Various maltodextrin-to-inulin ratios were tested, with the concentration of steviol glycosides kept constant at 2.5%. The study found that spray-dried steviol glycosides exhibited the most favorable physicochemical and sensory characteristics. FT-IR analysis confirmed that the chemical integrity of steviol glycosides was preserved during spray drying, and the reduction in hygroscopicity enhanced stability. Arriola et al. [74] investigated the encapsulation of *S. rebaudiana* aqueous leaf extract in sodium alginate beads and its impact on TPC and antioxidant stability (Figure 4a). Wet and lyophilized beads demonstrated high encapsulation efficiency (69.8% and 97.7%, respectively) and maintained TPC and antioxidant potential over 30 days at 4 °C. Lyophilization influenced bead size and morphology but effectively preserved polyphenols. Wet beads exhibited a smooth, spherical morphology (1.90 mm), while lyophilization reduced their size (1.24 mm) and introduced a spongy texture with cavities (Figure 4(bA,bB)). Confocal microscopy confirmed phenolic compound retention through red fluorescence emission (Figure 4(bC,bD)), supporting high encapsulation efficiency. 

**Table 2:** A comparative study of drying techniques, parameters, apparatus, and main observations for stevia (*S. rebaudiana*) leaves and extracts.

Compared Drying Methods	Equipment Specifications (Processing Scale)	Industrial Applicability (Industrial Use Cases)	Drying Conditions	Key Results	Ref.
Stevia leaves					
Spray, Freeze, and Vacuum oven drying	- Spray drying: Büchi Mini Spray Drier (B-191, Flawil, Switzerland; Continuous (lab to pilot-scale)) - Freeze drying: Christ Alpha 1-4 LD Plus (Batch; Osterode, Germany) - Vacuum oven drying: Heraeus Instruments Vacutherm VT 6025 (Batch; London, UK)	- Spray drying: High-throughput microencapsulation for functional ingredients (natural sweeteners, flavorings, beverage powders) - Freeze drying: Precision preservation of heat-sensitive compounds (nutraceutical powders, probiotic carriers, antioxidant-rich ingredients) - Vacuum oven drying: Gentle dehydration for prototype development (polyphenol stabilization, encapsulated botanical extracts, functional food trials)	- Spray drying: Inlet air temperature: 160 °C, Outlet air temperature: 88 °C, Feed temperature: 60 °C, Air pressure: 5 bar, Aspirator: 90%, Pump: 40%, Compressed air flow rate: 500 L/h - Freeze drying: Pressure: 0.017 mbar, Temperature: −57 °C, Time: 48 h - Vacuum pressure: <20 mbar, Temperature: 45 °C, Time: 2.5 h	- Spray-dried steviol glycosides had the best physicochemical and sensory properties, with reduced hygroscopicity and preserved chemical integrity - Samples dried by different methods showed significant differences in microencapsulation efficiency, moisture content, and solubility	[11]
Convective hot air, IR, and Vacuum drying	- Convective drying: Drying chamber: Stainless steel basket, Airflow: Constant airflow system, Hygro-Thermometer: Model 445 703, Extech Instrument Inc. (Batch; Waltham, MA, USA) - Vacuum cabinet dryer: Model VO400, Memmert GmbH (Batch; Schwabach, Germany) - IR radiation oven: Model HT490, Teka (Batch; Madrid, Spain)	- Convective drying: Energy-efficient dehydration of plant materials (herbs, teas, and food powders in small-scale processing) - IR drying: Rapid surface heating for delicate ingredients (botanical leaves, nutraceutical carriers, and pigment-rich materials) - Vacuum drying: Low-temperature preservation of sensitive compounds (polyphenol-rich extracts, functional ingredients, and powders for supplements)	- Convective drying: Temperature: 40, 60, and 80 °C, Airflow: 1.0 m/s, Load density: 0.938 kg/m^2^, Inlet air humidity: 63%, Sample load: 15 g, Initial moisture: 351.01 g water/kg d.m., Drying time to reach the Equilibrium MC: 270–930 min - Vacuum drying: Pressure: 150 mbar, Temperature: 40 °C, 60 °C, and 80 °C, Time: Until constant weight, Sample load: 15 g, Initial moisture: 351.01 g water kg^−1^ d.m., Drying time to reach the Equilibrium MC: 270–930 min - IR drying: Temperature: 40 °C, 60 °C, and 80 °C, Time: Until constant weight, Sample load: 15 g, Initial moisture: 351.01 g water kg^−1^ d.m., Drying time to reach the Equilibrium MC: 270–930 min	- Convective drying at 80 °C exhibited the highest moisture diffusivity, better leaf structure preservation, and the lowest energy consumption with the highest efficiency	[66]
Hot air, Freeze, and Shade drying	- Convective dryer (Batch), Freeze dryer (Batch), Shade drying (Traditional batch)	- Hot air drying: Rapid thermal dehydration for plant materials (herbs, teas, and polyphenol-enriched powders) - Freeze drying: High-retention preservation of sensitive bioactives (nutraceuticals, functional sweeteners, antioxidant-rich powders) - Shade drying: Low-cost, natural drying for artisanal processing (rural herbal products, teas, and traditional botanical remedies)	- Shade drying at 20 °C for 30 days - Hot air drying at 100 °C and 180 °C for 3 min in a convective drier - Freeze drying at a vacuum pressure of 9.5 × 10^−1^ mm Hg for 24 h	- The most suitable drying method was hot air at 180 °C, as it significantly increased total phenols, flavonoids, and antioxidants compared to fresh stevia leaves	[71]
Freeze, Convective, Vacuum, Microwave, IR, Sun, and Shade drying	- Freeze drying: Virtis Benchtop 3 L Freeze Dryer, Gardiner (Batch) (Gardiner, NY, USA) - Convective drying: Hot air dryer (Batch; Universidad de La Serena, Chile) - Vacuum drying: Memmert Vacuum Oven VO 400 (Batch; Frankfurt, Germany) - Microwave drying: IRT Microwave Oven MWM2812 W (Batch; Santiago, Chile) - IR drying: Teka HT490 IR Radiation Dryer (Batch; Velen, Germany), - Sun drier (Traditional batch) - Shade drier (Traditional batch)	- Freeze drying: High-retention preservation of sensitive bioactives (nutraceuticals, functional sweeteners, antioxidant-rich powders) - Convective drying: Energy-efficient dehydration of plant materials (herbs, teas, and food powders in small-scale processing) - Vacuum drying: Low-temperature preservation of sensitive compounds (polyphenol-rich extracts, functional ingredients, and powders for supplements) - Microwave drying: Rapid moisture removal for lab-scale applications (anti-inflammatory botanical extracts, functional plant powders) - IR drying: Surface-focused thermal drying (used for antimicrobial-enhanced powders and leaf-based nutraceuticals) - Sun drying: Traditional open-air drying (used in small-scale rural processing of herbs and teas) - Shade drying: Low-temperature, passive drying (preserves polyphenols in artisanal herbal and functional food products)	- Freeze drying: Initial freezing: −18 °C for 10 h, Pressure: 0.125 mbar, Temperature: −50 °C, Time: 10 h, - Convective drying: Temperature: 60 °C, Air velocity: 1.5 m/s, Time: ~3 h. - Vacuum drying: Temperature: 60 °C, Vacuum pressure: 15 kPa, Time: 4 h - Microwave drying: Power: 800 W, Sample weight: 50–80 g, Time: 4–8 min - IR drying: Temperature: 60 °C, Time: 3 h, Sample agitation: Every 30 min - Sun drying: Temperature range: 38.5 °C to 58.5 °C, Air humidity range: 11.5% to 53.5%, Time: Until constant weight is reached (variable drying time) - Shade drying: Temperature range: 25.5 °C to 30.1 °C, Air humidity range: 32% to 44.5%, Time: Until constant weight is reached	- Freeze drying and shade drying resulted in the highest polyphenol content and antioxidant capacity, while IR drying showed the lowest values. - Microwave drying and vacuum drying exhibited the strongest anti-inflammatory effects, and IR and convective drying showed prolonged antimicrobial activity.	[19]
Sun, Oven, and Microwave drying	- Oven (Batch; Model DGG-9070A, Shanghai, China) - Microwave: (Batch; Midea MG720FC8-NS, Foshan, China) - Sun drier (Traditional batch)	- Sun drying: Natural, cost-effective drying for low-tech settings (rural herbs, teas, and traditional botanical products) - Oven drying: Controlled thermal dehydration in lab-scale setups (fibrous plant materials, teas, and functional food prototypes) - Microwave drying: Rapid dielectric drying for small botanical loads (natural sweeteners, leaf extracts, and nutrient-retentive powders)	- Sun drying: Exposed to direct sunlight for about 5 days - Oven drying: Dried at 60 °C for 16 h - Microwave drying: Processed at 2450 MHz and 700 W for 6 min	- Stevia leaves maintained a high carbohydrate content (63.10–73.99%) across drying methods, supporting their use as a natural sugar substitute, with fiber remaining stable despite drying - Lead levels differed by drying method, with sun-dried leaves showing the highest concentration (4.77 μg/g) than oven (0.14 μg/g) and microwave (2.16 μg/g) drying	[69]
Sun, Oven, and Microwave drying	- Oven (Batch; Model DGG-9070A, Shanghai, China) - Microwave: (Batch; Midea MG720FC8-NS, Foshan, China) - Sun drier (Traditional batch)	- Sun drying: Natural, low-cost dehydration for traditional use (rural herbs, teas, and botanical materials with minimal equipment) - Oven drying: Uniform thermal drying under controlled heat (plant-based ingredients, teas, and fiber-rich materials for small-scale processing) - Microwave drying: Rapid, uniform drying with improved structural preservation (functional leaf powders, natural sweeteners, and volatile-sensitive compounds)	- Sun drying: Exposed to direct sunlight for about 5 days - Oven drying: Dried at 60 °C for 16 h - Microwave drying: Processed at 2450 MHz and 700 W for 6 min	- Drying method significantly influenced the structure, thermal stability, and volatile compound content of stevia leaves. - Microwave drying produced even, regular, and compact particles, while sun and oven drying resulted in angular brick-like particles.	[70]
Solar, Shade, Oven, and Microwave drying	- Microwave drier (Batch; Samsung Model ME731K, Kuala Lumpur, Malaysia) - A laboratory fan-oven dryer (Batch; Binder GmbH, Germany) - Shade drier (Traditional batch) - Solar drier (Traditional batch)	- Solar drying: Passive solar-assisted dehydration for low-cost applications (used in rural botanical and herbal product processing) - Shade drying: Low-temperature, non-invasive drying for quality retention (ideal for preserving glycosides in herbs and teas under artisanal conditions) - Oven drying: Controlled heat drying for balanced moisture removal (used in small-scale preparation of leaves, branches, and plant-based powders) - Microwave drying: Fast and uniform dehydration with effective moisture reduction (applied to functional leaves and sweetener-rich botanicals)	- Solar drying: Exposure to direct sunlight in open air at 44 °C for 24 h - Shade drying: Air-drying in a shaded area at 33 °C for 72 h - Microwave drier (Frequency: 2450 MHz, Power: 800 W, Time intervals of 1, 2, and 3 min) - Fan-oven drying (Temperatures: 40 °C, 50 °C, and 60 °C, Time for leaves: 24 h, Time for Branches: 48 h	- The highest glycoside content in leaves was achieved with solar drying (7.86%) and oven drying at 50 °C (7.84%). - Shade drying and microwave drying for 2 min produced the greatest dry matter in leaves, reaching 23.52% and 22.81%, respectively.	[14]
Radiation, Convection, Sun, and Shade drying	- Radiation drier (Batch) - Convection (Batch; stove was used for convection drying at 60 °C for the first two treatments) - Shade/Sun drier (Traditional batch)	- Radiation drying: Focused energy-based drying for bioactive retention (used in antioxidant-rich botanicals and phytochemical preservation) - Convection drying: Energy-efficient dehydration of plant materials (herbs, teas, and food powders in small-scale processing) - Sun drying: Natural, low-cost dehydration for traditional use (rural herbs, teas, and botanical materials with minimal equipment) - Shade drying: Low-temperature, passive drying for pigment and antioxidant retention (artisanal herbal products and functional botanicals)	- Convection drying: Stove temperature set at 60 °C, and leaves were weighed every 4 h - Shade/Sun drying: Temperature monitored at 29.7 °C and relative humidity at 70%, with leaves weighed every 24 h - The best time for radiation was 8 h - Final MC: 8.06% (Radiation), 7.45% (Convection), 6.97% (Sun), and 7.72% (Shade) drying	- Convection and shade drying preserved better color, higher pigment content, and enhanced antioxidant and ion chelation capacities	[72]
Oven, Sun, Microwave, and Freeze drying	- Not reported (Probably, in batch scale)	- Oven drying: Controlled heat-based dehydration for small-scale processing (used for teas, herbs, and functional food ingredients) - Sun drying: Cost-effective traditional drying method (applied in rural herbal processing and low-tech environments) - Microwave drying: Rapid, non-degradative drying for bioactive and sweetness preservation (suitable for phenolic-rich and sweetener compounds) - Freeze drying: Gentle drying for maximum retention of sensitive compounds (used in high-value nutraceuticals, stevia sweeteners, and antioxidant powders)	- Oven drying at 60 °C for 5 h and 80 °C for 3 h, microwave drying at 1200 W for 3 min, freeze drying the stevia leaves for 48 h, sunlight drying (direct sunlight for 12 h), final MC for all the methods: Until MC reach ≤20% of weight sample	- Microwave drying exhibited the highest DPPH radical scavenging activity and total phenolic content, comparable to freeze-dried and fresh leaves. - Microwave drying did not degrade stevioside or rebaudioside A content, maintaining the sweetening properties of stevia leaves	[15]
Oven, Shade, IR, Microwave, Sun, and Freeze drying	- Oven drying (Batch; GCA, Parsazma ovens, Tehran, Iran) - Freeze drying (Batch; Laboratory freeze dryer (VaCo 5-45, ZiRBUS, Bad Grund, Germany)) - IR drying (Batch; Electric IR drying machine) - Microwave drying (Batch; Samsung digital microwave (2450 MHz, 1000W, Kuala Lumpur, Malaysia) - Shade/Sun drier (Traditional batch)	- Oven drying: Controlled thermal drying for small-scale processing (preparation of plant-based powders, sweeteners, and herbal products) - Shade drying: Passive, low-heat method for preserving sensitive compounds (used in artisanal tea and botanical processing) - IR drying: Surface-directed drying for low-temperature applications (applied to delicate botanicals and glycoside-rich leaves) - Microwave drying: Short-duration dielectric heating for rapid moisture removal (suitable for bioactive-rich sweeteners and antioxidant powders) - Sun drying: Low-tech, cost-effective method with natural heat (used in rural herbal processing and traditional products) - Freeze drying: High-retention technique for thermosensitive ingredients (ideal for preserving steviol glycosides and antioxidant-rich extracts)	- Oven drying: with adjustable temperatures (45 ± 1 °C and 75 ± 1 °C) - Shade drying: 16–25 °C during the day - Freeze drying: Vacuum pressure: 9.1 × 10^−1^ mm Hg, Temperature: −55 °C - IR drying: Radiation temperature: 40 °C - Microwave drying: Duration: 60 s - Sun drying: Direct exposure for 36 h; ambient temperature varied with sunlight	- Freeze drying and sun-drying methods preserved the highest levels of steviol glycosides, including stevioside and rebaudioside A. - Oven, shade, IR, and microwave drying resulted in lower steviol glycoside content, with freeze drying and sun drying showing the least loss.	[75]
Oven and Shade drying	- Not reported (Probably, batch and traditional-batch, respectively)	- Oven drying: Controlled thermal drying with variable temperature impact (used in small-scale drying of botanical extracts, though it may reduce sensitive glycosides at high temperatures) - Shade drying: Gentle, passive dehydration method preserving sensitive compounds (used in artisanal teas, sweetener leaves, and traditional herb preparations)	- Oven drying: 60, 70, 80, and 90 °C for 6 h - Shade drying, 27 °C for 72 h	- Oven drying at 90 °C significantly increased stevioside, rebaudioside, and carbohydrate content, but reduced the rebaudioside/stevioside ratio. - Shade drying resulted in lower sweetener content than high-temperature oven drying and showed greater quality decline over storage.	[76]
**Stevia extracts**					
Spray drying and Electrospraying	- Spray dryer with dual fluid nozzle type (Continuous) - Electrospraying apparatus equipped with a variable high voltage 0–30 kV 160 power supply (Lab-/pilot-scale)	- Spray drying: Scalable encapsulation method for powdered bioactives (used in functional beverages, sweeteners, and phenolic-rich food powders) - Electrospraying: Mild, room-temperature technique for nano-/microencapsulation (applied to thermolabile bioactives, polyphenols, and targeted delivery in nutraceuticals)	- Spray drying: Using different flow rate (600–1000 mL/h), temperature (160 °C to 180 °C), maltodextrin: inulin concentration (5 and 10%) - Electrospraying: Using variable high voltage 0–30 kV 160 power supply at the room temperature	- Encapsulation efficiencies of 66.37% for spray drying and 67.47% for electrospraying were observed.	[65]
Sun, Shade, Air conditioner, and Oven drying	- All the drying instruments were on the batch laboratory scale.	- Sun drying: Low-cost natural drying (herbs, teas, botanicals) - Shade drying: Gentle drying for antioxidant retention (artisanal herbal products) - Air conditioner drying: Controlled low-temp drying (bioactives, antimicrobial compounds) - Oven drying: Thermal drying for small-scale use (functional powders, extracts)	- Sun drying: Average temperature (34.2 °C), Average humidity (52.12%), Sunshine duration (9.71 h/day) - Shade drying: Average temperature (26.05 °C), average humidity (66.21%), sunshine duration (0 h/day) - Air conditioner drying: Average temperature (22 °C), average humidity (49.3%), sunshine duration (0 h/day) - Oven drying: Average temperature (45 °C), average humidity (25%), sunshine duration (0 h/day)	- Air conditioning drying yielded the highest 2-tetradecyl acrylate content (25%) - Shade drying had the best anti-DPPH activity (IC_50_ = 57.94 μg/mL), while oven drying excelled in ABTS-inhibition capacity (IC_50_ = 44.03 μg/mL) - All methods inhibited *S. aureus*, with air conditioning and oven drying showing the strongest effects. - Air conditioning drying showed the highest cytotoxicity on MDA-MB-231 (breast cancer cell line), while sun drying had the least effect	[73]

## 6. Recent Applications of Dried and Extracted Forms of Stevia in Food Products

### 6.1. Dairy and Emulsion-Based Functional Foods

Dried stevia leaf powders have been incorporated into yogurts, cheeses, and other processed dairy products due to their ability to enhance sweetness and improve the nutritional profile without adding calories. Their use in milk, however, is less commonly reported in the literature. These powders may also offer benefits for lactose-intolerant individuals and health-conscious consumers seeking sugar alternatives. Meanwhile, free and microencapsulated stevia extracts are more versatile and can be used in a broader range of dairy products, including lactose-free milk, to replace lactose while maintaining a desirable taste and texture. Al-Taweel et al. [29] evaluated the quality and safety of the impact of flavored milk after adding stevia. They reported stevia’s potent antibacterial properties, especially against *S. aureus*, *P. aeruginosa*, and *E. coli*, with the plant’s extracts proving notable inhibitory effects. When stevia powder was added to flavored milk at concentrations of 0.3 and 0.4 g/50 mL, it not only replaced sugar but also maintained the product’s microbial safety over ten days of refrigeration at 4 °C. Moreover, stevia enhanced the milk’s flavor and color, making it a healthier alternative to sugar without compromising quality or safety. The addition of stevia to low-fat yogurt (0.03% *w*/*w*) and high-protein yogurt (0.04% *w*/*w*) was evaluated by Campos et al. [77]. Stevia-sweetened high-protein yogurts showed superior gel structure and rheological properties compared to other sweeteners, such as sucrose or agave. Sensory evaluations revealed that consumers preferred stevia-sweetened low-protein yogurts over those sweetened with sucrose or agave [77]. Bilgiç and Seyrekoğlu have recently developed a novel yogurt formulation by incorporating *S. rebaudiana* as a natural sweetener with 5% apple powder to evaluate its effects on physicochemical, rheological, and sensory properties during storage. Yogurts sweetened with 10–15% stevia not only maintained desirable water-holding capacity and structural integrity but also led to high consumer acceptance in terms of overall liking and purchase intent. Among the tested formulations, the sample containing 15% stevia had the highest dry matter content and water-holding capacity (67.73%) on day 20, while also showing a significant reduction in serum separation compared to the control. Although a decrease in pH and an increase in acidity were observed in all yogurt samples during storage, the combination of stevia and apple powder contributed to moderating these changes, suggesting a synergistic effect. Sensory evaluations also revealed that the yogurts containing stevia performed comparably or better than their sugar-sweetened counterparts, especially in the early storage phase [78].

Hameed et al. [79,80] investigated the antidiabetic potential of functional yogurts incorporating *Cinnamomum verum* and *S. rebaudiana*, using complementary in vitro and in vivo approaches. In their 2024 study, Hameed et al. demonstrated that yogurt formulations made from various milk sources, especially camel milk, showed significant α-amylase inhibitory activity, peaking at 61.15% on day 14, along with favorable changes in protein and ash content, pH, and acidity during storage [79]. Meanwhile, Hameed et al. [80] evaluated the antidiabetic efficacy of camel milk yogurt enriched with the same bioactives in streptozotocin-induced diabetic rats. Their results revealed significant reductions in fasting blood glucose and HbA1c levels, improved insulin sensitivity, and enhanced lipid metabolism, confirming the synergistic therapeutic effects of the cinnamon–stevia combination. These findings suggest that functional yogurt enriched with natural plant-derived compounds holds promise as both a preventive and therapeutic dietary intervention for type II diabetes. In another study [81], yogurt was enriched with instant stevia powder (0.1 to 0.4 g/100 mL) to evaluate its effects on quality parameters over 14 days of storage at 4 °C. The instant stevia powder, produced via microwave-assisted extraction and spray drying, improved the yogurt’s total phenolics and antioxidant capacity as well as textural attributes, with the lowest syneresis (17.09 g/100 g) observed at 0.4 g/100 mL ISP. However, sensory acceptability declined at concentrations above 0.2 g/100 mL. Overall, the study confirmed that yogurt fortification with instant stevia powder is an effective strategy for developing bioactive-rich dairy products with maintaining storage stability [81].

Mohammadi et al. [82] and Gharibzahedi and Altintas [83] both highlighted the multifunctional benefits of plant essential oils in dairy systems. Mohammadi et al. found that essential oils from *Ziziphora tenuior*, *Ferulago angulata*, and especially *Bunium persicum* (10%) enhanced the oxidative stability and sensory quality of traditional Iranian animal oil [82]. Similarly, Gharibzahedi and Altintas showed that tarragon essential oil, rich in methyl chavicol, improved the texture, stability, and shelf life of non-fat yogurt gels when delivered as a nanoemulsion, also enhancing sensory appeal and microbial resistance [83]. Based on this strategy, Evanuarini and Nidhal designed a low-calorie mayonnaise by substituting sugar with stevia leaf flour at different concentrations (0.1–0.5%). The incorporation of stevia leaf flour not only enhanced the nutritional profile, improving protein, ash, and carbohydrate content, but also significantly improved sensory attributes such as aroma, texture, and color. At 0.5% stevia leaf flour, the formulation resulted in the most favorable emulsion droplet stability and panelist acceptability, positioning stevia leaf flour as a promising natural sweetener and functional ingredient in emulsion-based products [84]. Overall, stevia as a viable sucrose alternative remarkably improved the texture and organoleptic scores of low- and high-protein yogurts and low-calorie mayonnaise.

### 6.2. Baked, Confectionary, and Snack Products

Incorporating stevia leaf or extract powders into confectionery and baked products provides a natural, low-calorie sweetening solution, contributing to a healthier product formulation without diminishing sweetness or sensory qualities. In this regard, Chughtai et al. [22] showed the potential of *S. rebaudiana* as a zero-calorie bio-sweetener with notable health benefits. Stevia cookies were created by substituting sucrose with stevia powder and extracts, showing promising results in rat models. The stevia-enriched diets effectively reduced blood glucose levels, increased insulin levels, and improved lipid profiles by lowering cholesterol, low-density lipoprotein (LDL), and triglycerides while boosting high-density lipoprotein (HDL). The administration of these stevia cookies did not adversely affect liver, renal, or hepatological parameters, confirming their safety. These findings support the inclusion of stevia not just for its sweetness but also for its potential health benefits in cookie formulations. Ali et al. [85] realized the potential of substituting sugar with stevia leaf in biscuit manufacturing to maintain sensory and storage qualities. Biscuits were prepared using stevia leaf powder and fresh juice as sugar substitutes and then tested for consumer preferences and organoleptic changes. Biscuits made with 18 g of stevia powder and 55 mL of stevia juice per kg of flour showed the best sensory results. Both types of stevia-based biscuits remained microbiologically safe and maintained their sensory qualities for one month when stored in glass, plastic jars, or tin cans. Armaya et al. [86] developed a low-sugar dried candied product from Padang Sidempuan snake fruit by replacing conventional sweeteners with stevia leaf powder (0.20–0.35%) and optimizing drying time (4–10 h). The formulation with 0.35% stevia and 8 h drying yielded the best physicochemical and sensory outcomes, showing reduced moisture and microbial counts, increased sugar and solid content, and favorable sensory attributes without adverse effects. These results demonstrated stevia’s potential as a natural sweetener in traditional fruit-based snacks.

### 6.3. Syrups, Drinks, Jams, and Preserves

Incorporating stevia leaf or extract powders into syrups, jams, and preserves delivers a healthier calorie-free sweetness without altering texture or flavor. Chranioti et al. [8] confirmed that spray drying-assisted microencapsulation is an effective method to decrease the bitter aftertaste of steviol glycosides in syrups. Sensory evaluation showed that the formulation with 2.5% steviol glycosides in total solids achieved the highest overall acceptance, presenting a balanced sweetness without exceeding the bitterness threshold. The encapsulated products displayed desirable properties, including improved stability due to reduced hygroscopicity. Abo Hashem et al. [87] also assessed the impact of incorporating cloves and cinnamon essential oils and 50% replacement in the sugar content with *S. rebaudiana* aqueous extract on the quality of mango and strawberry jam during 6 months of storage. These additions not only extended the shelf life of jams but also increased ash content and vitamin C levels without affecting the jams’ sensory qualities. Using 0.5 mL of essential oils per kilogram of jam, along with stevia extract, enhanced the jams’ nutritional value and longevity without any negative taste effects. Furthermore, Pielak et al. [88] replaced sugar with steviol glycosides in apple preserves with and without additional gelling agents or acidity regulators. They showed that a 10% substitution of sugar with steviol glycosides (0–0.05 g/100 g) maintained consumer satisfaction, while larger amounts (up to 40%, 0.20 g/100 g) with pectin and citric acid led to acceptable sensory quality. However, excessive substitution also led to undesirable flavor and odor changes. Physicochemical tests confirmed that low-sugar apple preserves with sugar substitution maintained good technological quality and sensory appeal. Earlier, Karagöz and Demirdöven [89] found that the chitosan-based edible coatings containing dried stevia leaf extract could successfully preserve fresh-cut apples with lower polyphenoloxidase activity, higher antioxidant capacity, and reduced microbial counts compared to control and chitosan-only coated samples. Wirivutthikorn [90] has recently assessed the use of foam mat drying to produce powdered drinks from a blend of pumpkin, carrot, and lemongrass juices, using egg albumen and carboxymethyl cellulose as foaming agents, and stevia syrup (0–50 mL) as a sweetener. The addition of stevia syrup significantly affected foam properties, with lower syrup concentrations leading to reduced foam density but increased foam stability, overrun, and yield. The lowest stevia level resulted in the best performance, with high foam stability (72.25%), overrun (80.59%), and product yield (15.29%). Although most sensory attributes remained unaffected by stevia levels, taste was significantly influenced. The resulting powder met microbiological safety standards and showed favorable physicochemical characteristics, suggesting that foam mat drying with stevia and protein–polysaccharide stabilizers is a promising method for producing stable, nutritious powdered beverages.

### 6.4. Fish and Meat Preservation

Kundu et al. [91] have recently assessed the effects of stevia extracts (1%, 2%, and 3%) obtained from leaves air-dried at room temperature (27–31 °C) for 3–4 days on the quality and shelf life of catla (*Gibelion catla*) fillets refrigerated for 20 days. They showed that the addition of 2% extract led to better preservation of chemical quality by significantly reducing pH, peroxide value, free fatty acids, thiobarbituric acid reactive substances (TBARS), and total volatile basic-nitrogen (TVB-N)) levels compared to other treatments. It also suppressed bacterial growth more effectively, with lower counts of aerobic plate bacteria, psychrotrophic bacteria, and other harmful microorganisms. Sensory evaluation revealed that fillets treated with 2% and 3% stevia extract retained better sensory attributes for up to 16 days, surpassing the control. Overall, 2% stevia extract was the optimal concentration for extending the shelf life and maintaining the quality of catla fillets during refrigeration [91].

The effectiveness of *S. rebaudiana* stem waste extract has been recently reported to retard lipid oxidation in salted–dried Pacific saury fillets during chilled storage. Yu et al. [92] explained that this extract, at a concentration of 0.2%, was superior to vitamin C in inhibiting lipid oxidation, as evidenced by lower peroxide values, TBARS, conjugated dienes, and acid values. The better efficacy of this extract was attributed to its higher Fe^2+^-chelating ability, despite having lower DPPH and ABTS radical-scavenging capacities compared to vitamin C. Liquid chromatography–mass spectrometry (LC-MS) profiling analysis identified potential functional phenolic compounds within the extract, reinforcing its potential as a natural antioxidant for preserving the quality of salted–dried fish products. Liu et al. [93] reported that *S. rebaudiana* leaf and stem extracts significantly reduced lipid oxidation in salted Pacific saury fillets with lower levels of peroxide, conjugated diene, and TBARS values. Interestingly, the leaf extract showed stronger lipid oxidation inhibition than the stem extract, despite having lower overall antioxidant activities, emphasizing its effectiveness in hydroxyl radical scavenging during the fish drying process.

Adding stevia glycosides could extend the shelf life of braised pork while maintaining critical quality parameters [94]. These researchers pointed out that the inclusion of stevia glycosides contributed to reducing lipid oxidation during cooking, resulting in a lower TBARS value compared to traditional white sugar. Furthermore, stevia glycosides enhanced the meat’s color and helped the formation of antioxidant peptides, which improved the nutritional value of the braised pork. As a result, stevia glycosides, as a natural sweetener, not only preserved the quality of meat but also improved its flavor and nutritional properties.

## 7. Conclusions and Future Prospectives

This overview shows that *S. rebaudiana* is rich in bioactive compounds, especially steviol glycosides like stevioside and rebaudioside A, which are responsible for its sweetness and potential health benefits, including anti-inflammatory, antioxidant, and antibacterial effects, as well as improvements in blood glucose and insulin sensitivity. The variability in these healthy functional constituents owing to different cultivation conditions indicates the complexity and therapeutic potential of stevia. The choice of drying technology profoundly impacts the preservation of stevia’s bioactive compounds. Solar drying is cost-effective but may compromise quality, while IR drying, if optimized, preserves key bioactive ingredients effectively. Hot air drying, though economical, can degrade sensitive components, whereas microwave drying ensures speed and uniformity, maintaining high levels of steviol glycosides and antioxidants. Vacuum oven drying is a gentler approach but comes with higher costs and complexity. Drying methods of spray drying, freeze drying, and microwave drying present better control over drying conditions and efficiency than traditional methods. Among these, spray drying can provide superior physicochemical and sensory qualities for stevia extracts, enhancing their functional properties while minimizing bitterness. Stevia’s diverse applications in food products, such as dairy, confectionery, and preserved items, demonstrate its ability to enhance sweetness and nutritional profiles while maintaining sensory quality and microbiological safety. The integration of stevia not only decreases sugar content but also improves the overall nutritional value and extends the shelf life of final food products.

To advance the practical applications and health benefits of *S. rebaudiana*, future research should prioritize several targeted areas. First, standardizing extraction and quantification protocols, such as HPLC and LC-MS-based glycoside profiling, is essential to ensure consistency in bioactive composition and therapeutic efficacy. This should be accompanied by investigations into the pharmacokinetics and long-term safety of lesser-studied steviol glycosides, like rebaudioside, through in vivo and clinical studies. Regarding drying technologies, future research should implement factorial design experiments (e.g., response surface methodology (RSM)) to systematically assess the effects of drying temperature, time, and air velocity on the retention of thermolabile compounds like stevioside, polyphenols, and flavonoids. Reproducibility challenges in stevia drying studies should be identified and addressed in future research via standardized reporting of critical parameters such as initial and final moisture content, drying time, and sample load. Comparative shelf-life studies assessing biochemical stability, microbial safety, and sensory retention in products dried via different methods (e.g., freeze, vacuum, or IR-assisted drying) are also warranted. Moreover, hybrid techniques, including IR-assisted solar drying and microwave-assisted vacuum drying, should be further evaluated for their scalability, energy consumption, and influence on key bioactive markers. Pilot-scale trials can help bridge the gap between laboratory innovation and industrial application. In spray drying, parameter optimization should focus on specific variables such as inlet/outlet temperature, feed flow rate, and atomization speed, ideally guided by predictive modeling tools. Furthermore, identifying novel encapsulating agents (e.g., protein–polysaccharide conjugates, nanocellulose, etc.) and their effect on bio-compound stability, dispersibility, and controlled release behavior could improve product functionality. Real-time monitoring of drying kinetics using advanced tools (e.g., near-IR spectroscopy or thermal imaging) and assessing long-term storage stability under different conditions will be essential for industrial translation.

## Figures and Tables

**Table 1:** An overview of drying methods, equipment, conditions, and key findings for stevia (*S. rebaudiana*).

Drying Method	Equipment Specifications (Processing Scale)	Industrial Applicability (Industrial Use Cases)	Drying Conditions	Findings	Ref.
Solar drying	Direct drying (cabinet-type dryer) and indirect drying (tunnel-type) assisted by flat plate solar collectors (Mostly batch)	Small- to medium-scale applications in solar-rich regions (Herbal teas, rural medicinal herb drying)	- 210 min for natural convection and 300 min for forced convection - Natural convection drying: 42 °C and 44 °C with a maximum value of 53 °C - Forced convective drying: 36 °C and 40 °C with a maximum value of 45 °C - Initial moisture content (MC): ~80%, Final MC: not reported	- Indirect drying offered benefits like moderate drying times, better control of conditions, and improved protection from temperature effects compared to direct drying.	[7]
	A developed solar dryer of mixed mode forced convection type (MFSCD, pilot/batch scale but easily scalable to small–medium industrial), and open sun drying (OSD, simple trays/mats laid outdoors under direct sun; strictly batch, informal scale)	MFSCD (Efficiently dries herbal leaves, retains antioxidants and flavor, ideal for small herbal processing enterprises in sunny regions with limited grid access), OSD (traditionally used in rural or artisanal settings; low-cost but slow and lower quality preservation, less suitable for commercial food and nutraceutical production)	- 870 min for open sun drying and 330 min for forced convection - Air entering temperature to the chamber was between 42.1 °C and 62.5 °C. - Initial MC: ~80%, Final MC: 5.03%, Sample load: 100 g	- The MFSCD achieved an overall dryer efficiency of 33.5% and an average exergy efficiency of 59.1%. - Solar-dried samples had higher antioxidant and flavonoid content and better color preservation compared to OSD samples.	[12]
	Solar air collector: (Area: 2.5 m^2^ (2.5 m length × 1 m width), Material: Black galvanized sheet iron absorber, ordinary glass cover, Inclination: 30°, Single circulation, glazed; Centrifugal fan: (Flow rate: 0.084 m^3^/s (adjustable 0.0296 to 0.0889 m^3^/s), Power: 0.1 kW, 220 V, Theoretical air velocity: 1.7 m/s); Ventilation duct: (Power: 0.1 kW, Includes a double T for partial or total air recirculation); Thermo regulator: (Temperature range: 0–99 °C, Connected to PT100 platinum probe and 4 kW resistive heaters); Drying chamber: (Dimensions: 1.40 m (height) × 0.90 m (length) × 0.50 m (width), Includes 10 racks, uniform hot air distribution) (Pilot/batch, expandable to small industrial)	- Small-scale industrial drying (used in stevia, herbal, and spice processing; off-grid or eco-friendly setups).	- Air temperatures: 50, 60, 70, and 80 °C, - Air flow rates: 150 and 300 m^3^/h	- The effective moisture diffusivity of stevia leaves ranged from 5.07 × 10^−11^ to 3.14 × 10^−10^ m^2^/s, increasing with higher drying temperatures and air flow - Higher drying temperatures (80 °C) and air flow led to quality degradation, reducing chlorophyll, total phenolic, and flavonoid content, and causing microstructural deformation.	[44]
Far-IR radiation	Far-IR drying equipment (Batch)	Batch-scale phytoproduct drying (used in drying of herbs, tea leaves, and plant-based ingredients with structural sensitivity)	- Drying temperatures: 45 °C, 50 °C, 55 °C, 60 °C, and 65 °C - Irradiation height: 60 mm, 120 mm, 180 mm, 240 mm, and 300 mm - Initial MC: 77.55%, Final MC: 10%, Drying time: 75–285 min, Sample load: 40 g on each tray (30 cm × 30 cm)	- Far-IR drying technology enhanced the formation of micropores on both the surface and within the materials, leading to improved quality of the dried stevia.	[45]
Medium-and short-wave IR drying	Dryer with both medium-wave IR (MIR) and short-wave IR (SIR) emission lamps, MIR lamps: Three medium-wave IR lamps (2–4 μm), radiation power of 450 W, 225 W, and 225 W, SIR lamps: Three SIR lamps (0.75–2 μm), radiation power of 450 W, 450 W, and 225 W, Thermal sensor: ±0.1 °C accuracy, placed on the material’s top surface (Batch)	- Batch-scale thermal drying (used in processing of heat-sensitive plant materials, teas, and nutraceutical powders with improved efficiency and compound preservation)	- Sample thickness: 3 cm, Drying temperatures: 40, 50, 60, 70, and 80 °C, Radiation distance: 10 cm (for uniform heating), Radiation power: 450, 225, and 225 W - Initial MC: 35%, 40%, 45%, and 50%, Final MC: 10%, Drying time: 6 h, Sample load: 400 g	- Medium-and short-wave IR drying reduced drying time by 15.38% and better preserved steviol glycosides and chlorogenic acids than other methods. - MIR drying at 60 °C was the most suitable, offering the best balance of drying time, energy consumption, and quality.	[16]
IR-assisted continuous hybrid solar dryer	- IR-assisted continuous belt conveyor dryer with four floors. Hybrid heating system combining solar and gas water heaters. Water pump to deliver water to the gas–liquid heat exchanger (tube-fin type). Centrifugal blower for ambient air intake. Inverter (LS, SV040iG5-4, Anyang, Korea) for adjusting air velocity. Temperature and relative humidity sensors (Aosong, AM2301/DHT21, Guangzhou, China). Digital anemometer (Lutron Model YK-80AM, Taipei, Taiwan) to measure air velocity. IR lamps (250 W) power placed above the conveyor belts (Continuous)	- Continuous-scale hybrid drying (used in automated drying of herbs, leafy botanicals, and food powders; suitable for energy-efficient industrial processing)	- Inlet air temperatures: 30 °C, 40 °C, and 50 °C. - Inlet air velocities: 7 m/s, 8 m/s, and 9 m/s - IR lamp input powers: 0 W, 150 W, and 300 W. - Initial MC: 77.63%, Final MC: 9.46%, Drying time: 10 h, Sample load: 30 g	- Higher inlet air temperatures and IR lamp power significantly shortened stevia leaf drying times. - The ANN model was the most precise for predicting moisture ratio (R^2^ = 0.9995), indicating strong accuracy over ANFIS and mathematical models.	[5]
(Convective) hot air drying	- Drying chamber: Transparent plastic chamber with a perforated tray (0.35 m^2^). Motor-centrifugal fan: 1/20 hp motor, centrifugal fan with a maximum airflow of 570 m^3^/h. Heating system: Three finned resistances (1.5 kW each, totaling 4.5 kW). - Temperature control: PID (Proportional–Integral–Derivative) controller with PT 1000 thermocouple to regulate temperature. Air velocity control: Variable current transformer for fan speed regulation (Batch)	- Batch-scale convective drying (used in drying of herbs, tea leaves, and plant-derived ingredients; widely adopted in food and herbal industries)	- Air circulates longitudinally through the upper and lower parts of the drying tray. - The air is heated by the resistances and then enters the drying chamber. - Temperature (45 °C, 55 °C, and 65 °C) and air velocity (2, 3, and 4 m/s) are controlled and monitored during the drying process. - Initial MC: 81.58–83.40%, Final MC: 6.71–8.46%, Drying time: 240, 330, and 450 °C, Sample load: 20 g on each perforated tray	- The highest drying rate of 0.05 kg water/kg dry matter min was achieved at 65 °C and 4 m/s air velocity. - The Page model accurately fitted the drying data (R^2^ > 0.9994), with temperature and air velocity increasing ΔE (total color difference) and glucoside values.	[40]
	- Lab-scale 440 V convective dryer with temperature control and blower fan (Batch)	- Batch-scale convective drying (used in laboratory-scale dehydration of herbs, leaves, and botanical powders for quality optimization and model development)	- At 30–80 °C, 1 m/s air velocity, and 16% humidity, with weight checked every 10 min until stable. - Initial MC: 80.66%, Final MC: <10%, Drying time: 60–530 min, Sample load: 1200 g on each tray	- The ANFIS model (R^2^ = 0.9998) provided a more accurate prediction of drying kinetics for stevia leaves than ANN and mathematical models. - Higher drying temperatures reduced water activity, ascorbic acid, and antioxidant levels, while increasing water solubility, hygroscopicity, particle density, and flowability of the dried stevia powder.	[6]
	- A drying chamber is connected to an Interface System (Ohaus RS232, Parsippany, NJ, USA), which links the analytical balance to a computer (Batch)	- Batch-scale convective drying (used in precision drying of botanical samples for compositional and thermal sensitivity analysis)	- Temperature: 30, 40, 50, 60, 70, and 80 °C, Constant air velocity: 2.0 m/s - Initial sample weight: 80 g, Load density: 2.08 kg/m^2^, Refrigerated storage at 5.2 °C before drying - Initial MC: 76.64%, Final MC: 4.5–7.7%, Drying time: 570, 480, 270, 180, 120, and 60 min, Sample load: 80 g	- Vitamin C content in stevia leaves decreased with increasing drying temperature. - Phenolics and flavonoids increased below 50 °C, while sweeteners rose to 50 °C with no significant increase above it.	[17]
Microwave drying	A domestic microwave oven (IFB Industries, 900 W, 2450 MHz) with a rotating 30 cm glass plate (Batch-scale and lab- or pilot-level application)	- Batch-scale dielectric drying (used in rapid drying of small botanical samples; suitable for high-throughput lab screening of thermosensitive compounds)	- Microwave Power Levels: 180, 300, 450, 720, and 900 W, Sample Size: 25 g of stevia leaves per trial, Final moisture content target: 0.1 g/g (dry basis); Weighing interval: Defined time gaps with completion in under 10 s - Initial MC: 80.66%, Final MC: <10%, Drying time: 2.5–15 min, Sample load: g	- Microwave drying at 720 W preserved the maximum stevioside, rebaudioside A, and ascorbic acid content in stevia leaves. - At 900 W, stevia samples achieved peak total phenol content and antioxidant capacity, with effective diffusivity increasing alongside power levels.	[46]
Freeze drying	Freeze dryer (FDU-830, Tokyo Rikakikai Co., Ltd., Tokyo, Japan), a laboratory device which requires precise temperature control to prevent degradation of sensitive freeze-dried materials (Batch)	Batch-scale freeze drying (used in preservation of sensitive emulsions and bioactive-rich botanicals for nutraceutical and pharmaceutical applications)	- −40 °C for 30 min in a circulation bat, ~5 Pa for 24 h with/without trehalose to lyophilize microemulsions containing transglycosylated stevia (stevia-G)	- Freeze-dried, redispersible particles with trehalose form stable, gastrointestinal-soluble emulsions, enabling flavanone release via stevia-G-based submicron emulsions.	[47]
	Laboratory freeze dryer model Christ ALPHA 1–2 LD plus (Batch)	Batch-scale freeze drying (used in food-grade preservation of moisture-sensitive ingredients; applicable to powders for functional foods, nutraceutical blends, and encapsulated bioactives)	- 24 h, −50 °C, and 0.1 mbar, after being frozen overnight	- Freeze-dried stevia absorbed more water vapor compared to the other treatment (gamma irradiation).	[31]
	Ultra-freezer (NEW BRUNSWICK-U101, Hertfordshire, England), Laboratory freeze-dryer device (Labconco, Kansas, MO, USA) (Batch)	Batch-scale freeze drying (used in micro/nanoencapsulation of bioactive ingredients; applicable to functional food powders, sweetener systems, and flavor masking in nutraceutical formulations)	- Freezing at −56 °C for 6 h, Lyophilization at 0.110 mbar vacuum pressure, and −80 °C in the condenser	- The encapsulation efficiency reached 84.37%, with optimal sweetness release achieved at 5% sweetener and 5% wall material concentrations. - Micro- and nanocapsules had stable water activity (0.49) and solubility (17.65%), effectively masking the bitterness of stevia.	[48]
Vacuum oven drying	Vacuum oven (Batch)	- Batch-scale vacuum drying (used in the gentle dehydration of bioactive-rich formulations; applicable to encapsulated extracts, functional powders, and thermo-sensitive ingredients in food and supplement industries)	- Drying for 6 h at 50 °C, Initial MC of stevia before the extract preparation: 74.73%	- The best protection of bioactives was observed in the sample prepared with a 1:1 ratio of Arabic gum to maltodextrin, while the 1:3 ratio was the most preferred by the panelists.	[37]
Spray drying	A Mobile Minor concurrent flow spray dryer equipped with a pneumatic pulse rotary spray dryer (Continuous (pilot to industrial))	- Large-scale encapsulation and powder production (Functional food powders, natural sweeteners, plant-based beverages, instant mixes, and nutraceutical ingredients)	- Spray rate of 23,000 rpm, Heat-drying airflow of 84 kg/h - Lower conditions: Inlet air temperature (160 °C) and feed flow rate (2 kg/h); Higher conditions: Inlet air temperature (200 °C) and feed flow rate (3 kg/h)	- Influence of inlet air temperature and feed flow rate on phenol and flavonoid content, with higher temperatures reducing concentrations. - High feed flow rates aid in the preservation of flavonoids, making the final product a source of polyphenolic compounds.	[49]
	Not reported (Probably, continuous (lab to pilot-scale))	- Controlled encapsulation of sensitive compounds (stabilized bioactive powders, flavor carriers, and long-shelf-life functional ingredients)	- Feed rate: 15 mL/min, Inlet temperature: 120 °C	- The microencapsulation process achieved a high yield of 98.20%, with no significant change in particle size distribution during storage. - Storage temperature affected the physical stability, as changes in physical characteristics were observed over the one-month period.	[50]
	Mini Spray Dryer (Büchi, model B-191) (Continuous (lab-scale))	- High-efficiency microencapsulation for formulation testing (Prototype development of functional food ingredients, stevia-rich sweetener systems, and antidiabetic nutraceuticals)	- Shaker apparatus at 200 rpm, 35 °C for 30 min, Inlet temperature of 175 °C, Outlet temperature of 103 °C	- Microencapsulation efficiency reached 84%, enhancing solubility by threefold compared to the free fraction - SEM images showed spherical microcapsules with reduced moisture and increased hygroscopicity, facilitating stevia-rich phenolic incorporation in antidiabetic functional foods and beverages	[51]
	A laboratory spray dryer with a two-fluid nozzle (B-191, Büchi Mini Spray Drier, Flawil, Switzerland) (Continuous (lab-scale))	- Formulation optimization and sensory improvement (Sweetener syrups, encapsulated stevia extracts, and sensory-enhanced functional ingredients)	- Inlet air temperature of 160 °C, Outlet air temperature of 88 °C, Feed temperature: 60 °C, Air pressure of 5 bar, Aspirator of 90%, Pump (maximum capacity) of 40%, Compressed air flow rate of 500 L/h	- Higher steviol glycoside levels improved encapsulation efficiency, increased hygroscopicity, and slightly reduced solubility. - Syrups demonstrated stable viscosity, a consistent refractive index, and low turbidity, with the best sensory characteristics at 2.5% steviol glycosides	[8]
	Not reported (Probably, continuous (lab-scale))	- Color clarification and bioactive encapsulation (Natural sweetener production, pigment-reduced extracts, and shelf-stable stevia-based powders)	- Inlet air temperature: 160 °C, Water temperature outlet: 88 °C, Feed temperature: 60 °C, Air pressure: 5 bar, Aspirator: 90%, pump: 40%, Compressed air flow rate: 500 L/h	- Clarification of stevia extract using acid-activated kaolin successfully reduced 95% of green pigment and 65% of yellow pigment. - The optimal encapsulated stevia product contained 0.83% stevioside and 2.25% rebaudioside A, with 9.8% moisture content and 12.91% hygroscopicity.	[3]
	Encapsulator Büchi-B390 (Büchi Labortechnik AG, Flawil, Switzerland) with a nozzle size of 1000 μm (Batch)	- Batch-scale microencapsulation (Targeted delivery and controlled release systems, encapsulated polyphenols, gastro-resistant formulations, and functional food microspheres)	- Biopolymers dissolved in stevia (400 mL), Vibration frequency of 800 Hz, Pressure of 120 mbar	- Adding xanthan gum (XG) and casein (CA) to calcium alginate (ALG) microspheres improved encapsulation efficiency and controlled TPC release. - Composite microspheres (ALG + XG + CA) prevented degradation in simulated gastric fluids but fully released total polyphenols in intestinal fluids.	[52]

## Data Availability

Data is contained within the article.

## Abbreviations

The following abbreviations are used in this manuscript:

ADI	Acceptable Daily Intake
ALG	Alginate
ANN	Artificial Neural Network
ANFIS	Adaptive Neuro-Fuzzy Inference System
CA	Casein
DSC	Differential Scanning Calorimetry
FT-IR	Fourier Transform Infrared
GAE	Gallic Acid Equivalent
IR	Infrared
MFSCD	Mixed Mode Forced Convection Dryer
MLF	Multilayer Feed-forward
MSWID	Medium- and Short-Wave Infrared Drying
OSD	Open Sun Drying
SEM	Scanning Electron Microscope
SIR	Short-wave Infrared
MIR	Medium-wave Infrared
SVglys.	Steviol Glycosides
Tg	Glass Transition Temperature
TPC	Total Polyphenolic Content
XG	Xanthan Gum

## References

[1] World Health Organization (WHO) (2023). Diabetes Fact Sheet. https://www.who.int/news-room/fact-sheets/detail/diabetes.

[2] International Diabetes Federation (IDF) (2021). IDF Diabetes Atlas.

[3] Martono Y., Pranawati F.E.R., Riyanto C.A., Muninggar J. (2019). Clarification and Encapsulation of *Stevia rebaudiana* Bertoni Water Extract. J. Phys. Conf. Ser..

[4] Gharibzahedi S.M.T., Yousefi S., Chronakis I.S. (2019). Microbial Transglutaminase in Noodle and Pasta Processing. Crit. Rev. Food Sci. Nutr..

[5] Bakhshipour A., Zareiforoush H., Bagheri I. (2021). Mathematical and Intelligent Modeling of Stevia (*Stevia rebaudiana*) Leaves Drying in an Infrared-Assisted Continuous Hybrid Solar Dryer. Food Sci. Nutr..

[6] Kalsi B.S., Singh S., Alam M.S., Sidhu G.K. (2023). Comparison of ANN and ANFIS Modeling for Predicting Drying Kinetics of *Stevia rebaudiana* Leaves in a Hot-Air Dryer and Characterization of Dried Powder. Int. J. Food Prop..

[7] Castillo-Téllez M., Figueroa I.P., Téllez B.C., Vidaña E.C.L., Ortiz A.L. (2018). Solar Drying of Stevia (Rebaudiana Bertoni) Leaves Using Direct and Indirect Technologies. Sol. Energy.

[8] Chranioti C., Chanioti S., Tzia C. (2015). Microencapsulation of Steviol Glycosides (*Stevia rebaudiana* Bertoni) by a Spray Drying Method–Evaluation of Encapsulated Products and Prepared Syrups. Int. J. Food Stud..

[9] Gharibzahedi S.M.T., Smith B. (2021). Legume Proteins Are Smart Carriers to Encapsulate Hydrophilic and Hydrophobic Bioactive Compounds and Probiotic Bacteria: A Review. Compr. Rev. Food Sci. Food Saf..

[10] Gharibzahedi S.M.T., Jafari S.M., Jafari S.M. (2017). Nano-Capsule Formation by Cyclodextrins. Nanoencapsulation Technologies in the Food and Nutraceutical Industries.

[11] Chranioti C., Chanioti S., Tzia C. (2016). Comparison of Spray, Freeze and Oven Drying as a Means of Reducing Bitter Aftertaste of Steviol Glycosides (Derived from *Stevia rebaudiana* Bertoni Plant)—Evaluation of the Final Products. Food Chem..

[12] Lakshmi D.V.N., Muthukumar P., Layek A., Nayak P.K. (2019). Performance Analyses of Mixed Mode Forced Convection Solar Dryer for Drying of Stevia Leaves. Sol. Energy.

[13] Periche A., Castelló M.L., Heredia A., Escriche I. (2015). Influence of Drying Method on Steviol Glycosides and Antioxidants in *Stevia rebaudiana* Leaves. Food Chem..

[14] Al-Amrani H.A., Al-Taweel S.K., Abdul-Qader Z.M. (2018). Steviosides Composition in *Stevia rebaudiana* Bertoni: Effect of Drying Methods. Plant Arch..

[15] Abdul Halim A., Mohd Zain Z., Mubarak A., Tufail Ahmad F. (2019). Effect of Different Drying Methods on Antioxidant Properties, Stevioside and Rebaudioside A Contents of Stevia (*Stevia rebaudiana* Bertoni) Leaves. Asian J. Agric. Biol..

[16] Ai Z., Ren H., Lin Y., Sun W., Yang Z., Zhang Y., Zhang H., Yang Z., Pandiselvam R., Liu Y. (2022). Improving Drying Efficiency and Product Quality of *Stevia rebaudiana* Leaves Using Innovative Medium-and Short-Wave Infrared Drying (MSWID). Innov. Food Sci. Emerg. Technol..

[17] Lemus-Mondaca R., Ah-Hen K., Vega-Gálvez A., Honores C., Moraga N.O. (2016). *Stevia rebaudiana* Leaves: Effect of Drying Process Temperature on Bioactive Components, Antioxidant Capacity and Natural Sweeteners. Plant Foods Hum. Nutr..

[18] Muanda F.N., Soulimani R., Diop B., Dicko A. (2011). Study on Chemical Composition and Biological Activities of Essential Oil and Extracts from *Stevia rebaudiana* Bertoni Leaves. LWT-Food Sci. Technol..

[19] Lemus-Mondaca R., Vega-Gálvez A., Rojas P., Stucken K., Delporte C., Valenzuela-Barra G., Jagus R.J., Agüero M.V., Pasten A. (2018). Antioxidant, Antimicrobial and Anti-Inflammatory Potential of *Stevia rebaudiana* Leaves: Effect of Different Drying Methods. J. Appl. Res. Med. Aromat. Plants.

[20] JECFA (2008). Joint FAO/WHO Expert Committee on Food Additives. Steviol glycosides. Compendium of Food Additive Specifications.

[21] Morissette A., de Wouters d’Oplinter A., Andre D.M., Lavoie M., Marcotte B., Varin T.V., Trottier J., Pilon G., Pelletier M., Cani P.D. (2024). Rebaudioside D Decreases Adiposity and Hepatic Lipid Accumulation in a Mouse Model of Obesity. Sci. Rep..

[22] Chughtai M.F.J., Pasha I., Ahsan S., Mehmood T., Khalid M.Z., Farooq M.A., Liaqat A., Khaliq A., Tanweer S., Ungureanu-Iuga M. (2024). Metabolic Syndrome Extenuation in Rat Model by Feeding *Stevia rebaudiana* Bertoni Cookies. Cogent Food Agric..

[23] Han J.-Y., Park M., Lee H.-J. (2023). Stevia (*Stevia rebaudiana*) Extract Ameliorates Insulin Resistance by Regulating Mitochondrial Function and Oxidative Stress in the Skeletal Muscle of Db/Db Mice. BMC Complement. Med. Ther..

[24] Alavala S., Nalban N., Sangaraju R., Kuncha M., Jerald M.K., Kilari E.K., Sistla R. (2020). Anti-Inflammatory Effect of Stevioside Abates Freund’s Complete Adjuvant (FCA)-Induced Adjuvant Arthritis in Rats. Inflammopharmacology.

[25] Mehmood A., Althobaiti F., Zhao L., Usman M., Chen X., Alharthi F., Soliman M.M., Shah A.A., Murtaza M.A., Nadeem M. (2022). Anti-Inflammatory Potential of Stevia Residue Extract Against Uric Acid-Associated Renal Injury in Mice. J. Food Biochem..

[26] Qiao G., Ji W., Sun Z., Wang X., Li P., Jia H., Duan L., Qi F. (2022). Isosteviol Reduces the Acute Inflammatory Response after Burns by Upregulating MMP9 in Macrophages Leading to M2 Polarization. Int. Immunopharmacol..

[27] Farid A., Hesham M., El-Dewak M., Amin A. (2020). The Hidden Hazardous Effects of Stevia and Sucralose Consumption in Male and Female Albino Mice in Comparison to Sucrose. Saudi Pharm. J..

[28] Shahu R., Kumar D., Ali A., Tungare K., Al-Anazi K.M., Farah M.A., Jobby R., Jha P. (2023). Unlocking the Therapeutic Potential of *Stevia rebaudiana* Bertoni: A Natural Antiglycating Agent and Non-Toxic Support for HDF Cell Health. Molecules.

[29] Al-Taweel S.K., Al-Anbari I.H., Al-Hamdani H.M. (2022). Antioxidant identification, antimicrobial activity of *Stevia rebaudiana* Bertoni leaves extract on flavored milk. Int. J. Agric. Stat. Sci..

[30] Barczewski M., Aniśko J., Hejna A., Mysiukiewicz O., Kosmela P., Sałasińska K., Boczkowska A., Przybylska-Balcerek A., Stuper-Szablewska K. (2023). Ground Lemon and Stevia Leaves as Renewable Functional Fillers with Antioxidant Activity for High-Density Polyethylene Composites. Clean Technol. Environ. Policy.

[31] Hidar N., Noufid A., Abouloifa H., Mouhib M., Asehraou A., Jaouad A., Mahrouz M. (2023). Comparative Study of Two Preservation Methods (Freeze Drying and Gamma Irradiation) on the Phenolic Profile, Antioxidant and Antimicrobial Activities of the Essential Oils of Stevia. J. Food Meas. Charact..

[32] Lahijanian S., Eskandari M., Akhbarfar G., Azizi I., Afazel M., Ghobadi C. (2023). Morphological, Physiological and Antioxidant Response of *Stevia rebaudiana* Under In Vitro Agar Induced Drought Stress. J. Agric. Food Res..

[33] Lremizi I., Ait Ouazzou A., Bensouici C., Fauconnier M.-L. (2023). Chemical Composition, Antioxidant, Anticholinesterase, and Alpha-Glucosidase Activity of *Stevia rebaudiana* Bertoni Extracts Cultivated in Algeria. J. Food Meas. Charact..

[34] Sansano S., Rivas A., Pina-Pérez M.C., Martinez A., Rodrigo D. (2017). *Stevia rebaudiana* Bertoni Effect on the Hemolytic Potential of *Listeria monocytogenes*. Int. J. Food Microbiol..

[35] Shahu R., Jobby R., Patil S., Bhori M., Tungare K., Jha P. (2022). Phytochemical Content and Antioxidant Activity of Different Varieties of *Stevia rebaudiana*. Hortic. Environ. Biotechnol..

[36] Xu Q., Liu M., Chao X., Zhang C., Yang H., Chen J., Zhou B. (2023). Stevioside Improves Antioxidant Capacity and Intestinal Barrier Function While Attenuating Inflammation and Apoptosis by Regulating the NF-Κb/MAPK Pathways in Diquat-Induced Oxidative Stress of IPEC-J2 Cells. Antioxidants.

[37] Fishi A.N.A., Nurhadi B., Mahani, Saputra R.A. (2023). The Effect of Arabic Gum and Maltodextrin on the Physicochemical Properties of Vacuum-Dried Stevia (*Stevia rebaudiana* Bertoni) Extract Powder. Food Res..

[38] Chakma A., Afrin F., Rasul M.G., Maeda H., Yuan C., Shah A.K.M.A. (2023). Effects of Extraction Techniques on Antioxidant and Antibacterial Activity of Stevia (*Stevia rebaudiana* Bertoni) Leaf Extracts. Food Chem. Adv..

[39] Alshawwa S.Z., Mohammed E.J., Hashim N., Sharaf M., Selim S., Alhuthali H.M., Alzahrani H.A., Mekky A.E., Elharrif M.G. (2022). In Situ Biosynthesis of Reduced Alpha Hematite (A-Fe_2_O_3_) Nanoparticles by *Stevia rebaudiana* L. Leaf Extract: Insights Into Antioxidant, Antimicrobial and Anticancer Properties. Antibiotics.

[40] Castillo-Téllez B., Téllez M.C., López-Vidaña E.C., Domínguez Niño A., Mejía-Pérez G.A., Vega-Gómez C.J. (2023). Temperature–Air Velocity Association, Experimental and Modeling Study of Stevia Leaves Solar Drying. Energy Explor. Exploit..

[41] Darwish N.N., Karkor E.M.M.A., Abou Tahoun A.M., Attia A.S. (2024). Effect of Drying Methods and Storage Packages on Some Stevia Varieties Quality Traits. Al-Azhar J. Agric. Res..

[42] Ahmadi R., Kalbasi-Ashtari A., Gharibzahedi S.M.T. (2012). Physical Properties of Psyllium Seed. Int. Agrophys..

[43] Taheri-Garavand A., Nassiri A., Gharibzahedi S.M.T. (2012). Physical and Mechanical Properties of Hemp Seed. Int. Agrophys..

[44] Hidar N., Ouhammou M., Mghazli S., Idlimam A., Hajjaj A., Bouchdoug M., Jaouad A., Mahrouz M. (2020). The Impact of Solar Convective Drying on Kinetics, Bioactive Compounds and Microstructure of Stevia Leaves. Renew. Energy.

[45] Huang X., Li W., Wang Y., Wan F. (2021). Drying Characteristics and Quality of *Stevia rebaudiana* Leaves by Far-Infrared Radiation. LWT.

[46] Kalsi B.S., Singh S., Alam M.S., Bhatia S. (2023). Microwave Drying Modelling of *Stevia rebaudiana* Leaves Using Artificial Neural Network and Its Effect on Color and Biochemical Attributes. J. Food Qual..

[47] Uchiyama H., Asai S., Nakanishi A., Tandia M., Kadota K., Tozuka Y. (2022). Applicability of Transglycosylated Stevia for Oil-in-Water Submicron Emulsions by High-Pressure Homogenization. Food Sci. Technol. Res..

[48] Micanquer-Carlosama A., Serna-Cock L., Ayala-aponte A. (2017). Double Emulsion and Complex Coacervation in Stevia Encapsulation. Vitae.

[49] Chaparro-Hernández I., Méndez-Lagunas L., Rodríguez-Ramírez J., Sandoval-Torres S., Áquino-González L., Barriada-Bernal G. (2023). Spray drying of *Stevia rebaudiana* Bertoni Aqueous Extract: Effect on Polyphenolic Compounds. Chem. Eng. Transac..

[50] Mutmainah M., Martono Y., Franyoto Y.D., Kusmita L. (2019). Microencapsulation Stability of Stevia Leaf Extracts of *Stevia rebaudiana* Bert Using Inulin-Chitosan. Open Access Libr. J..

[51] Zorzenon M.R.T., Hodas F., Milani P.G., Formigoni M., Dacome A.S., Monteiro A.R.G., Mareze-Costa C.E., Costa S.C. (2019). Microencapsulation by Spray-Drying of Stevia Fraction with Antidiabetics Effects. Chem. Eng. Trans..

[52] Kolar S., Jurić S., Marijan M., Vlahoviček-Kahlina K., Vinceković M. (2022). Applicability of Alginate-Based Composite Microspheres Loaded with Aqueous Extract of *Stevia rebaudiana* Bertoni Leaves in Food and Pharmaceutical Products. Food Biosci..

[53] Kumar P., Tripathy P.P. (2024). Experimental and Computational Analysis of Drying Characteristics and Quality Attributes of Indirect and Mixed-Mode Solar Dried *Stevia rebaudiana* Leaves. J. Food Process Eng..

[54] Kumar P., Rani P., Tripathy P.P. (2025). Indirect Mode Solar Drying of Stevia (*Stevia rebaudiana*) Leaves: Experimental and Numerical Investigation of Fluid Flow Pattern, Moisture and Temperature Distribution Profile. Biomass Convers. Biorefin..

[55] Gharibzahedi S.M.T., Razavi S.H., Mousavi M. (2013). Kinetic Analysis and Mathematical Modeling of Cell Growth and Canthaxanthin Biosynthesis by *Dietzia natronolimnaea* HS-1 on Waste Molasses Hydrolysate. RSC Adv..

[56] Bassey E.J., Cheng J.-H., Sun D.-W. (2022). Improving Drying Kinetics, Physicochemical Properties and Bioactive Compounds Of Red Dragon Fruit (*Hylocereus species*) by Novel Infrared Drying. Food Chem..

[57] Zhang Y., Zhu G., Li X., Zhao Y., Lei D., Ding G., Ambrose K., Liu Y. (2020). Combined medium-and short-wave infrared and hot air impingement drying of sponge gourd (*Luffa cylindrical*) slices. J. Food Eng..

[58] Alizadeh Bahaabad G., Gharibzahedi S.M.T., Esmaiili M., Alizadeh M. (2014). Response Surface Modeling for Optimization of Textural and Color Characteristics of Dried Grapes. Int. J. Food Eng..

[59] Askari Vaselabadi S., Gharibzahedi S.M.T., Greiner R., Vale J.M., Ovenseri A.C., Rashidinejad A., Roohinejad S. (2025). Advancements in Spray-Drying for the Microencapsulation of Fat-Soluble Vitamins: Stability, Bioavailability and Applications. J. Food Biochem..

[60] Kakoei H., Mortazavian A.M., Mofid V., Gharibzahedi S.M.T., Hosseini H. (2023). Single and Combined Hydrodistillation Techniques of Microwave and Ultrasound for Extracting Bio-Functional Hydrosols from Iranian *Eryngium caucasicum* Trautv. Chem. Pap..

[61] Golbargi F., Gharibzahedi S.M.T., Zoghi A., Mohammadi M., Hashemifesharaki R. (2021). Microwave-Assisted Extraction of Arabinan-Rich Pectic Polysaccharides from Melon Peels: Optimization, Purification, Bioactivity and Techno-Functionality. Carbohydr. Polym..

[62] Rostami H., Gharibzahedi S.M.T. (2017). Mathematical Modeling of Mucilage Extraction Kinetic from the Waste Hydrolysates of Fruiting Bodies of *Zizyphus jujuba* Mill. J. Food Process. Preserv..

[63] Nurhad B., Andoyo R., Indiarto R. (2012). Study the Properties of Honey Powder Produced from Spray Drying and Vacuum Drying Method. Int. Food Res. J..

[64] Mitra J., Shrivastava S.L., Rao P.S. (2015). Non-enzymatic browning and flavour kinetics of vacuum dried onion slices. Int. Agrophys..

[65] Oikonomopoulou V., Stramarkou M., Plakida A., Krokida M. (2022). Optimization of Encapsulation of Stevia Glycosides Through Electrospraying and Spray Drying. Food Hydrocoll..

[66] Lemus-Mondaca R., Zura-Bravo L., Ah-Hen K., Di Scala K. (2021). Effect of Drying Methods on Drying Kinetics, Energy Features, Thermophysical and Microstructural Properties of *Stevia rebaudiana* Leaves. J. Sci. Food Agric..

[67] Yang H.I., Ameer K., Chung Y.B., Min S.G., Eun J.B. (2023). Optimization of Spray Drying Process for Recovery of Onion–Stevia Leaf Hot Water Extract Powder Using Response Surface Methodology. Food Sci. Nutr..

[68] Hidar N., Noufid A., El Adnany E.M., Lahnine L., Idlimam A., Mouhib M., Jaouad A., Mahrouz M. (2023). Sorption Behavior and Thermodynamic Characteristics of Stevia Leaves as Affected by Freeze Drying and Gamma Irradiation Technologies. Euro-Mediterr. J. Environ. Integr..

[69] Gasmalla M.A.A., Yang R., Amadou I., Hua X. (2014). Nutritional Composition of *Stevia rebaudiana* Bertoni Leaf: Effect of Drying Method. Trop. J. Pharm. Res..

[70] Gasmalla M.A.A., Tessema H.A., Alahmed K., Hua X., Liao X., Yang R. (2017). Effect of Different Drying Techniques on the Volatile Compounds, Morphological Characteristics and Thermal Stability of *Stevia rebaudiana* Bertoni Leaf. Trop. J. Pharm. Res..

[71] Periche A., Castelló M.L., Heredia A., Escriche I. (2016). Effect of Different Drying Methods on the Phenolic, Flavonoid and Volatile Compounds of *Stevia rebaudiana* Leaves. Flavour Fragr. J..

[72] Moguel-Ordóñez Y.B., Cabrera-Amaro D.L., Segura-Campos M.R., Ruiz-Ruiz J.C. (2015). Studies on Drying Characteristic, Nutritional Composition and Antioxidant Properties of *Stevia rebaudiana* (Bertoni) Leaves. Int. Agrophys..

[73] Özyiğit Y., Esra U., Eruygur N., Mehmet A., İnanir M., Halil B.A.L., Kahrizi D., Turgut K. (2023). Comparison of Different Drying Methods for Phytochemical Quality of Stevia (*Stevia rebaudiana* Bert.). Not. Sci. Biol..

[74] Arriola N.D.A., de Medeiros P.M., Prudencio E.S., Müller C.M.O., Amboni R.D.D.M.C. (2016). Encapsulation of Aqueous Leaf Extract of Stevia Rebaudiana Bertoni with Sodium Alginate and Its Impact on Phenolic Content. Food Biosci..

[75] Karimi M., Direkvand-moghadam F., Sheibani N. (2023). Variation in Steviol Glycoside Contents of Stevia (*Stevia rebaudiana*) Leaves Under Various Leaf Drying Processes. Iran. J. Plant Physiol..

[76] Zalat M., Abido A.I., Radwan F.I., Zeitoun A.A., Shaaban E.H. (2019). Drying Temperature and Storage Period and Their Relation to Stevia Leaf Chemical Composition. J. Adv. Agric. Res..

[77] Campos L., Tuma P., Silva T., Gomes D., Pereira C.D., Henriques M.H. (2024). Low Fat Yoghurts Produced with Different Protein Levels and Alternative Natural Sweeteners. Foods.

[78] Bilgiç İ.G., Seyrekoğlu F. (2025). The Use of Stevia (*Stevia rebaudiana*) as a Sweetener in Fruit Yogurts Produced with Apple Powder and the Determination of Quality Parameters. J. Food Meas. Charact..

[79] Hameed A., Ashraf F., Anwar M.J., Amjad A., Hussain M., Imran M., Mujtaba A., Ahmad I., Aslam M.S., El-Ghorab A.H. (2024). α-Amylase Enzyme Inhibition Relevant to Type II Diabetes by Using Functional Yogurt with *Cinnamomum verum* and *Stevia rebaudiana*. Food Agric. Immunol..

[80] Hameed A., Ishtiaq F., Zeeshan M., Akhtar S., Ismail T., Ahmad R.S., Amir M., Anwar M.J. (2023). Combined Antidiabetic Potential of Camel Milk Yogurt with *Cinnamomum verum* and *Stevia rebaudiana* by Using Rodent Modelling. J. Food Sci. Technol..

[81] Akalan M., Bayrak Akay K., Başyiğit B., Karakuş M.Ş., Yücetepe M., Karaaslan A., Karaaslan M. (2024). Instant Stevia Powder as a Novel Potential Additive for Enhancing Nutritional Value and Quality Characteristics of Yogurt. J. Food Sci. Technol..

[82] Mohammadi M., Khorshidian N., Gharibzahedi S.M.T. (2021). The Extended Oxidative and Sensory Stability of Traditional Dairy-Based Oil with Steam-Distilled Essential Oils Extracted from the Bioactive-Rich Leaves of *Ziziphora tenuior*, *Ferulago angulata*, and *Bunium persicum*. J. Food Qual..

[83] Gharibzahedi S.M.T., Altintas Z. (2022). Transglutaminase-Induced Free-Fat Yogurt Gels Supplemented with Tarragon Essential Oil-Loaded Nanoemulsions: Development, Optimization, Characterization, Bioactivity and Storability. Gels.

[84] Evanuarini H., Nidhal H.A. (2023). The Potential of Stevia Leaf Flour (*Stevia rebaudiana*) as a Natural Sweetener in Mayonnaise. Food Sci. Technol..

[85] Ali A., Saha K.K., Choudhury S., Islam N. (2022). Formulation, Senso-Chemical Analysis and Shelf-Life Study of Biscuits Using Stevia Leaf as the Substitute for Sugar. Asian J. Dairy Food Res..

[86] Armaya R.P., Lubis L.M., Nurminah M. (2024). The Effect of the Amount of Stevia Leaf Powder (*Stevia rebaudiana*) and Drying Time on the Quality of Snake Fruit Padang Sidempuan Dried Candied. Indones. J. Agric. Res..

[87] Abo Hashem M.M., Nassar A.G., Abdelhamied A.A., Sulaiman A.M. (2023). Effect of Addition Essential Oils and Partial Substituting of Sugar with Stevia Leaves Aqueous Extract on the Quality of Both Mango and Strawberry Jam During Storage. Arch. Agric. Sci. J..

[88] Pielak M., Czarniecka-Skubina E., Głuchowski A. (2020). Effect of Sugar Substitution with Steviol Glycosides on Sensory Quality and Physicochemical Composition of Low-Sugar Apple Preserves. Foods.

[89] Karagöz Ş., Demirdöven A. (2019). Effect of Chitosan Coatings with and Without Stevia Rebaudiana and Modified Atmosphere Packaging on Quality of Cold Stored Fresh-Cut Apples. LWT.

[90] Wirivutthikorn W. (2025). Effects of Stevia Syrup on Powdered Drink Products from Pumpkin Carrot and Lemongrass by Foam Mat Drying. Curr. Res. Nutr. Food Sci. J..

[91] Kundu A., Chakma A., Dulal M.A., Rasul M.G., Mondal M.N., Shah A.A. (2024). Effects of Stevia (*Stevia rebaudiana* Bertoni) Leaf Extracts on the Quality and Shelf Life of Refrigerated Catla (*Gibelion catla*) Fillets. J. Agric. Food Res..

[92] Yu H., Liu W., Zhou X., Lv H., Nakano T., Liu H., Zhao Q., Yang G. (2024). Effect of *Stevia rebaudiana* Stem Waste Extract on Lipid Oxidation of Salted-Dried Pacific Saury During Chilled Storage. LWT.

[93] Liu H., Yang G., Zhao Q., Li H., Niu L., Wu H., Yu H. (2022). Antioxidant Effects of *Stevia rebaudiana* Leaf and Stem Extracts on Lipid Oxidation in Salted Pacific Saury (*Cololabis saira*) During Processing. Eur. J. Lipid Sci. Technol..

[94] He Z.G., Zhang Y., Yang M.D., Zhang Y.Q., Cui Y.Y., Du M.Y., Zhao D., Sun H. (2022). Effect of Different Sweeteners on the Quality, Fatty Acid and Volatile Flavor Compounds of Braised Pork. Front. Nutr..

